# The modified Polsby–Popper score, a novel quantitative histomorphological biomarker and its potential to predict lymph node positivity and cancer‐specific survival in oral tongue squamous cell carcinoma

**DOI:** 10.1002/cam4.6824

**Published:** 2023-12-22

**Authors:** Tamás Dániel Csűry, Anna Zsófia Csűry, Matthias Balk, Andreas M. Kist, Robin Rupp, Sarina K. Mueller, Matti Sievert, Heinrich Iro, Markus Eckstein, Antoniu‐Oreste Gostian

**Affiliations:** ^1^ Department of Otolaryngology, Head & Neck Surgery University Hospital Erlangen, Friedrich‐Alexander‐Universität Erlangen‐Nürnberg Erlangen Germany; ^2^ Comprehensive Cancer Center EMN University Hospital Erlangen, Friedrich‐Alexander‐Universität Erlangen‐Nürnberg Erlangen Germany; ^3^ Bavarian Cancer Research Center (Bayerisches Zentrum für Krebsforschung, BZKF) Erlangen Germany; ^4^ Independent Scholar; ^5^ Department Artificial Intelligence in Biomedical Engineering Friedrich‐Alexander‐Universität Erlangen‐Nürnberg Erlangen Germany; ^6^ Institute of Pathology University Hospital Erlangen, Friedrich‐Alexander‐Universität Erlangen‐Nürnberg Erlangen Germany

**Keywords:** computer‐assisted image analysis, squamous cell carcinoma of head and neck, survival analysis, tongue cancer

## Abstract

**Background:**

The significance of different histological spreading patterns of tumor tissue in oral tongue squamous cell carcinoma (TSCC) is well known. Our aim was to construct a numeric parameter on a continuous scale, that is, the modified Polsby–Popper (MPP) score, to describe the aggressiveness of tumor growth and infiltration, with the potential to analyze hematoxylin and eosin‐stained whole slide images (WSIs) in an automated manner. We investigated the application of the MPP score in predicting survival and cervical lymph node metastases as well as in determining patients at risk in the context of different surgical margin scenarios.

**Methods:**

We developed a semiautomated image analysis pipeline to detect areas belonging to the tumor tissue compartment. Perimeter and area measurements of all detected tissue regions were derived, and a specific mathematical formula was applied to reflect the perimeter/area ratio in a comparable, observer‐independent manner across digitized WSIs. We demonstrated the plausibility of the MPP score by correlating it with well‐established clinicopathologic parameters. We then performed survival analysis to assess the relevance of the MPP score, with an emphasis on different surgical margin scenarios. Machine learning models were developed to assess the relevance of the MPP score in predicting survival and occult cervical nodal metastases.

**Results:**

The MPP score was associated with unfavorable tumor growth and infiltration patterns, the presence of lymph node metastases, the extracapsular spread of tumor cells, and higher tumor thickness. Higher MPP scores were associated with worse overall survival (OS) and tongue carcinoma‐specific survival (TCSS), both when assessing all pT‐categories and pT1‐pT2 categories only; moreover, higher MPP scores were associated with a significantly worse TCSS in cases where a cancer‐free surgical margin of <5 mm could be achieved on the main surgical specimen. This discriminatory capacity remained constant when examining pT1‐pT2 categories only. Importantly, the MPP score could successfully define cases at risk in terms of metastatic disease in pT1‐pT2 cancer where tumor thickness failed to exhibit a significant predictive value. Machine learning (ML) models incorporating the MPP score could predict the 5‐year TCSS efficiently. Furthermore, we demonstrated that machine learning models that predict occult cervical lymph node involvement can benefit from including the MPP score.

**Conclusions:**

We introduced an objective, quantifiable, and observer‐independent parameter, the MPP score, representing the aggressiveness of tumor growth and infiltration in TSCC. We showed its prognostic relevance especially in pT1‐pT2 category TSCC, and its possible use in ML models predicting TCSS and occult lymph node metastases.

## Graphical abstract

We established an objective, numeric parameter, that is, the MPP score, reflecting the aggressiveness of tumor spread in tongue squamous cell carcinoma, with the potential to analyze hematoxylin and eosin‐stained whole slide images in an automated manner. The MPP score showed an excellent prognostic potential in terms of survival prediction. We showed its possible use in machine learning models predicting occult cervical lymph node metastases as well.

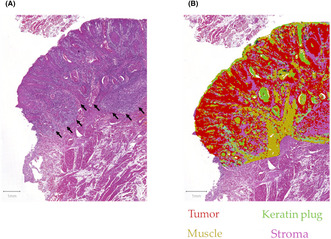

## 1 INTRODUCTION

Head and neck squamous cell carcinoma (HNSCC) represents more than 90% of all head and neck malignancies and is well known for its heterogeneity, with a broad spectrum of fine histological features and subsite‐specific characteristics.^1^, ^2^ Among HNSCCs, squamous cell carcinoma of the mobile tongue (TSCC) accounted for an estimated number of 17,860 new cancer cases in the United States in 2022.^3^ It has been reported by several authors to have an increasing incidence, especially in young White individuals.^2^, ^4^ While Kim et al. emphasized the excellent survival of appropriately treated TSCCs according to statistical analyses of the Surveillance, Epidemiology, and End Results (SEER) database, other recent reports have shown a modest 5‐year disease‐free survival of 63% across all T‐categories.^5^, ^6^ Moreover, tumor recurrence occurs in up to 30% of early stage TSCCs.^7^ Considering these epidemiological and survival characteristics, health care providers may face emerging challenges in treating a growing number of TSCC patients in an individual risk‐adapted approach.^5^ According to international guidelines, surgery (management of the primary tumor and cervical lymph nodes) constitutes the cornerstone of the treatment, followed by adjuvant radio(chemo)therapy based on defined clinicopathologic features.^8^ However, important aspects of the surgical therapy remain controversial.

Considering surgical margins, the *conditio sine qua non* of an adequate R0, that is, a microscopically margin‐negative resection is that the tumor specimen be surrounded by at least 5‐mm‐wide cancer‐free mucosal tissue. However, the effect of different free margin widths on oncological outcomes in the context of different histopathologic features has not been fully elucidated. Hakim et al. investigated 753 oral squamous cell carcinoma (OSCC) cases without regard to histopathologic risk factors other than the resection margin status and showed that T1‐T2 tumors resected with a safety margin of >5 mm and 1–4 mm have comparable survival outcomes without benefiting from adjuvant therapy, while larger tumors with a safety margin of <5 mm benefit from postoperative radiation.^9^ Notably, Brandwein‐Gensler et al. established a histological risk assessment of OSCC in 2005, which was based partly on the worst pattern of invasion (WPOI) of the tumor at the cancer/host interface. Their investigations brought several noteworthy conclusions: (1) the histological risk assessment proved to be a better prognosticator of overall and relapse‐free survival than surgical free margins, (2) tumors with free margins and a high risk score could benefit from adjuvant radiation even in the absence of perineural spread, and (3) low‐ or intermediate‐risk tumors might benefit more from additional resection of the primary site than from adjuvant radiotherapy.^10^ Nonetheless, WPOI is included in the reporting system of the College of American Pathologists solely as a “redundant” and “optional” parameter, whereas WPOI‐5 only (tumor dispersion ≥1 mm between tumor satellites) is regarded as relevant.^1^, ^11^

The management of the clinically negative neck is another issue that warrants further evaluation. Elective neck dissection is indicated in patients with clinical suspicion of metastatic cervical lymph nodes and in cT3‐cT4a cancers; however, according to the guidelines of the European Society for Medical Oncology (ESMO), head and neck specialists can choose a sentinel lymph node biopsy in T1‐T2 cancers if the depth of invasion (DOI) is less than 10 mm; moreover, for DOI <5 mm and cT1N0, active surveillance of the neck is regarded as a valid treatment option.^8^ Nevertheless, there is a crucial need for a more exact prediction of possible cervical nodal metastases, as neck dissection may cause severe surgical morbidities^12^; however, regional relapse has a substantially negative impact on survival.

The adverse effects of several tumor features on the oncological outcome, including pathological T‐category, cervical lymph node involvement, DOI, perineural spread of tumor cells (PNI), and lymphovascular invasion (LVI), are well‐documented.^13^, ^14^, ^15^, ^16^, ^17^, ^18^ However, conventional histological grading is known not to correlate well with clinical prognosis.^19^ Machine learning algorithms have been developed, for example, to predict occult node metastases in OSCC.^20^, ^21^ However, these models have a limited input in terms of features characteristic of the intrinsic biological behavior of OSCC and do not take into account the pattern of invasion (POI), although a significant association between POI and nodal status, a strong predictor of OSCC, has been described.^10^ Moreover, PNI and LVI, which proved to possess a high discriminatory potential, have been reported to be present in approximately 20%–40% and 10%–30% of the OSCC cases, respectively.^6^, ^21^, ^22^, ^23^ This implies that in more than half of the OSCC cases, an additional descriptor of tumor aggressiveness would be of utmost importance, and that this descriptor should ideally be interpreted on a continuous scale, as converting inherently non‐categorical into categorical data will potentially result in loss of information through a priori assumptions.^24^

The approach to diagnosing and confirming cancer through microscopic examination of resected tissue has remained largely unchanged since the late 1800s.^25^ With the advent of high‐throughput digital scanning of standard histopathology glass slides, computational pathology was born and the term pathomics was coined “to embody the wide variety of data that are captured from image analyses to generate quantitative features to characterize the diverse phenotypic features of tissue samples in whole slide images (WSIs)”.^26^ The pathomics approach is particularly useful in revealing features and relationships that are not directly interpretable by the naked eye and in reducing interobserver variability. In our investigation, we employ the term “pathomics” specifically to describe the extraction of explainable, quantitative features from histological structures through large‐scale data mining based on WSIs.^27^ More specifically, we propose a quantitative variable describing tumor growth and infiltration to be interpreted on a continuous numeric scale using the armamentarium of image analysis and evaluated by its relation to survival. We investigate its interplay with resection margin status and demonstrate its possible use for machine learning models that predict survival and lymph node positivity.

## 2 MATERIALS AND METHODS

### 2.1 Study population

The investigated cohort consisted of patients with pT1‐pT4a squamous cell carcinomas of the mobile tongue (TSCC) who underwent curative surgical therapy at the Department of Otorhinolaryngology and Head and Neck Surgery of the University Hospital Erlangen between 2007 and 2019. We avoided sampling bias by randomly selecting digitized TSCC histopathology slides first without any knowledge of patient information or oncological outcomes. Only after completing the image analysis pipeline of the study (see below) did we begin to retrieve information from complete medical records, including patient demographics, clinicopathologic features, interventions, and outcomes, in a comprehensive, retrospective manner. Further patient parameters, such as smoking status, socioeconomic status, and other potential confounding factors, were not collected, as they were beyond the scope of this investigation. Tumor staging was performed according to the Union for International Cancer Control (UICC) staging system, 8th edition. All cases were rereviewed by a board‐certified pathologist during cohort composition (ME).

### 2.2 Computer‐assisted processing and analysis of whole slide histopathology images

**FIGURE 1:**
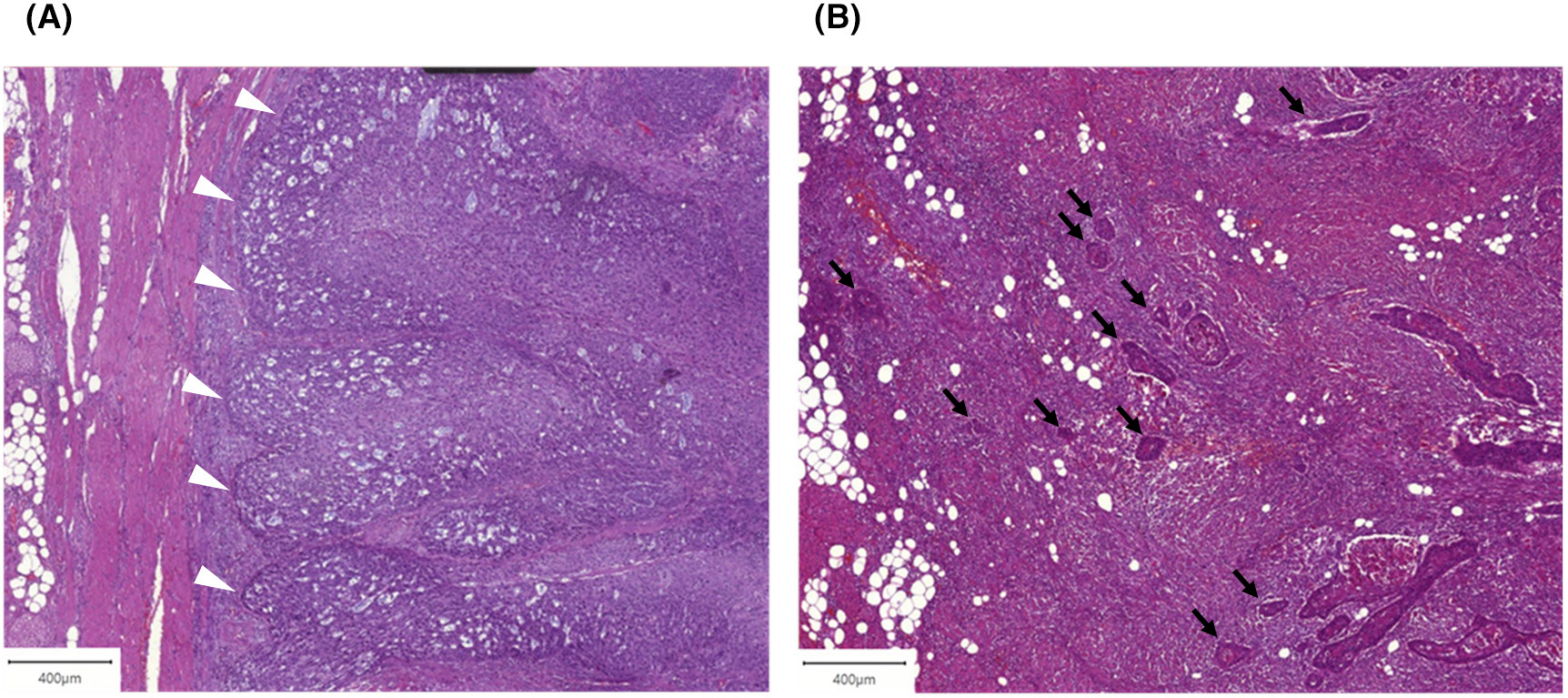
Examples of (A) compact and (B) discontinuous infiltration patterns of tongue squamous cell carcinoma (TSCC), hematoxylin and eosin (H&E) staining. White arrowheads: infiltration front of a tumor with compact infiltration pattern. Black arrows: disjoint tumor islands on the infiltration front of a tumor with discontinuous infiltration pattern.

A total of 100 formalin‐fixed paraffin‐embedded (FFPE) primary tumor specimens of resected TSCC were examined under expert guidance by a board‐certified pathologist with knowledge in head and neck pathology (author ME) who provided continuous supervision throughout the study. Hematoxylin and eosin (H&E) staining was performed on sections of each FFPE sample. Glass slides were digitized with the Pannoramic 1000 Flash Scanner (3DHISTECH Ltd, Budapest, Hungary) resulting in whole slide images (WSI); each of the 100 WSIs belonged to a separate patient. The open‐source digital pathology software QuPath Version 0.3.2 was used for all further image manipulation and analysis.^28^ All analyses were performed in a collaborative effort between the first author, a resident with a 4‐year background in image analysis, and the abovementioned pathologist with expertise in head and neck pathology. Both experts reviewed and revised all the cases in consensus to ensure consistency and accuracy. First, we assessed the growth and infiltration patterns of each tumor in a qualitative, dichotomous manner, as previously described by Eckstein et al., without any knowledge of the clinical outcome.^29^ Briefly, a cohesive growth pattern signified tumor cells spreading in the form of larger cell nests, whereas a discohesive growth pattern signified tumors consisting of smaller disjoint tumor cell islands. A compact infiltration pattern denoted tumors with an infiltrative front separating the main tumor mass from the adjacent parenchyma in an unequivocal manner (Figure 1A). The invasive front was characterized as discontinuous when no distinct invasive front could be delineated because of scattered tumor cell islands in a wider area of the surrounding tongue parenchyma (Figure 1B). The presence of one tumor cell island at a distance of 1 mm from the main tumor mass was considered sufficient to assign this category to a WSI. Moreover, we measured the tumor thickness on each WSI as the entire dimension of the tumor from the surface to the point of the deepest invasion.^30^

We then applied a semiautomatic image analysis pipeline to each of the WSIs (a step‐by‐step description along with visual examples can be downloaded as Supplementary Material 1). First, we marked the tumor in its entirety on the WSI with a sufficient amount of adjacent healthy tongue parenchyma, the region of interest (ROI), thus consisting of a central and peritumoral area. An experienced pathologist (author ME) estimated the percentage of the stromal component in the central tumor area with the naked eye:

(1) $$\frac{\text{Estimated stromal component}}{\text{Estimated stromal component}+\text{estimated tumor component}}\times 100$$

**FIGURE 2:**
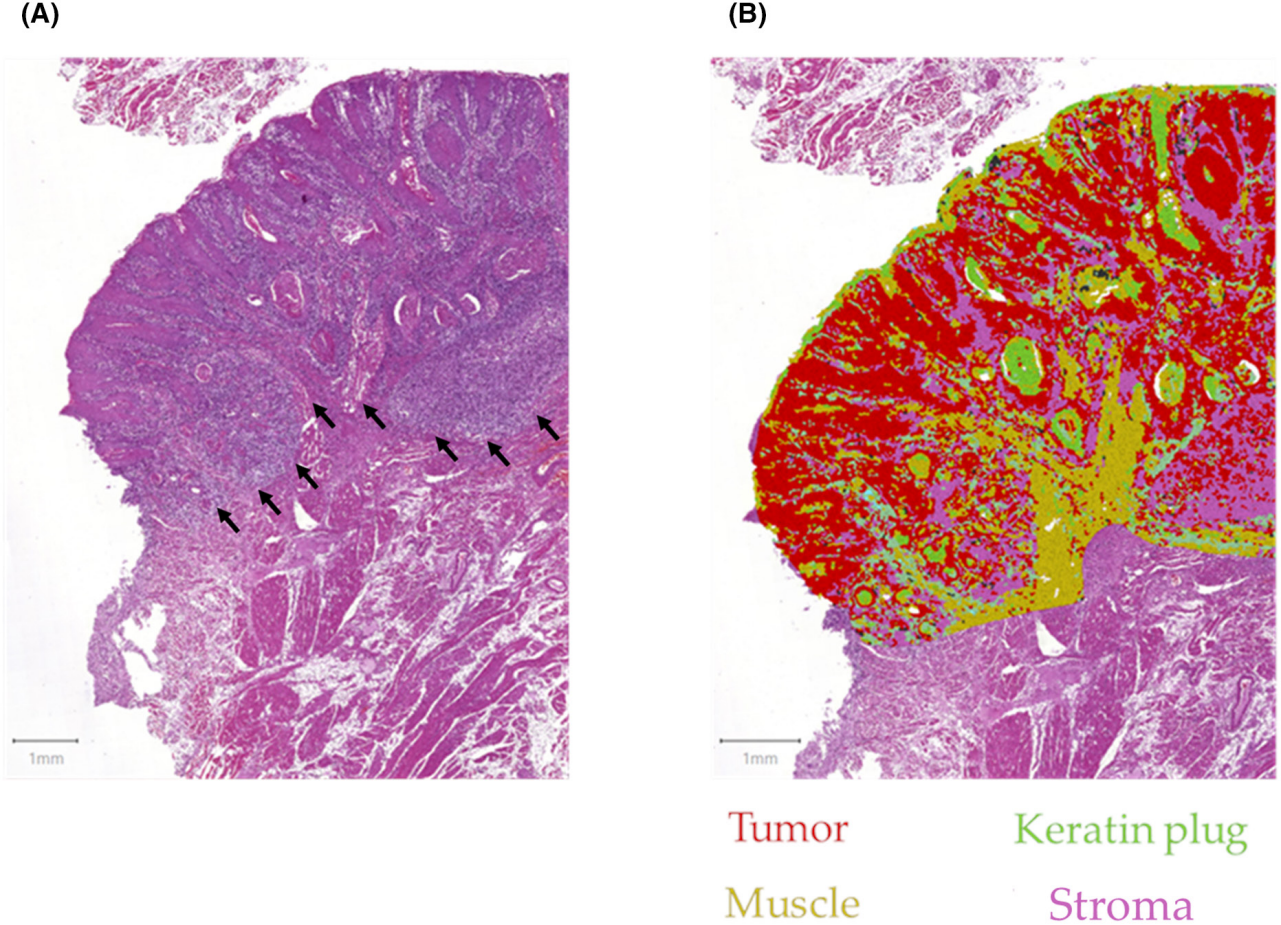
Examples of a result of the semiautomated tissue classification pipeline: (A) area of a hematoxylin and eosin (H&E)‐stained tongue squamous cell carcinoma whole slide image to be analyzed with the tumor front marked with black arrows (B) end result with different colors representing different tissue compartments. The architecture of the tumor and its spatial relationship with different tissue compartments can be clearly observed with an acceptable signal/noise‐ratio.

Subsequently, as an image preprocessing step, we applied superpixel segmentation to the ROI. Superpixels are perceptually meaningful atomic regions of an image.^31^ In the present study, this step was chosen to help reduce computational demand; moreover, we found that ROIs segmented in this way were easy to interpret after computer‐aided superpixel classification had been performed, resembling a pathologist's mental process evaluating subsegments of a slide under the microscope. Next, intensity features were computed for each superpixel. We then performed superpixel classification in an iterative, visually supervised manner by providing the software example regions of different tissue compartments (tumor, fibrous stroma, lymphocytic stroma, muscle, etc.) until sufficient accuracy of tissue detection regarding the whole ROI was reached, that is, the architecture of the tumor and its relationship to other tissue compartments was clearly recognizable on the processed images (Figure 2).

Superpixels of the same class were then merged into annotations, whose geometric parameters (most importantly, perimeter and area) could be exported using a custom script (to be downloaded at https://github.com/annesophie96/HNSCC‐scripts/tree/main/scripts). The percentage of the stromal component in the central tumor area based on computer‐assisted tissue detection was calculated as follows:

(2) $$\frac{\text{Area of the stromal component}}{\text{Area of the stromal component}+\text{area of the tumor component}}\times 100,$$

and the results were compared with the pathologist*'*s estimates for quality control.

### 2.3 Developing a numeric feature describing tumor growth and infiltration pattern

**FIGURE 3:**
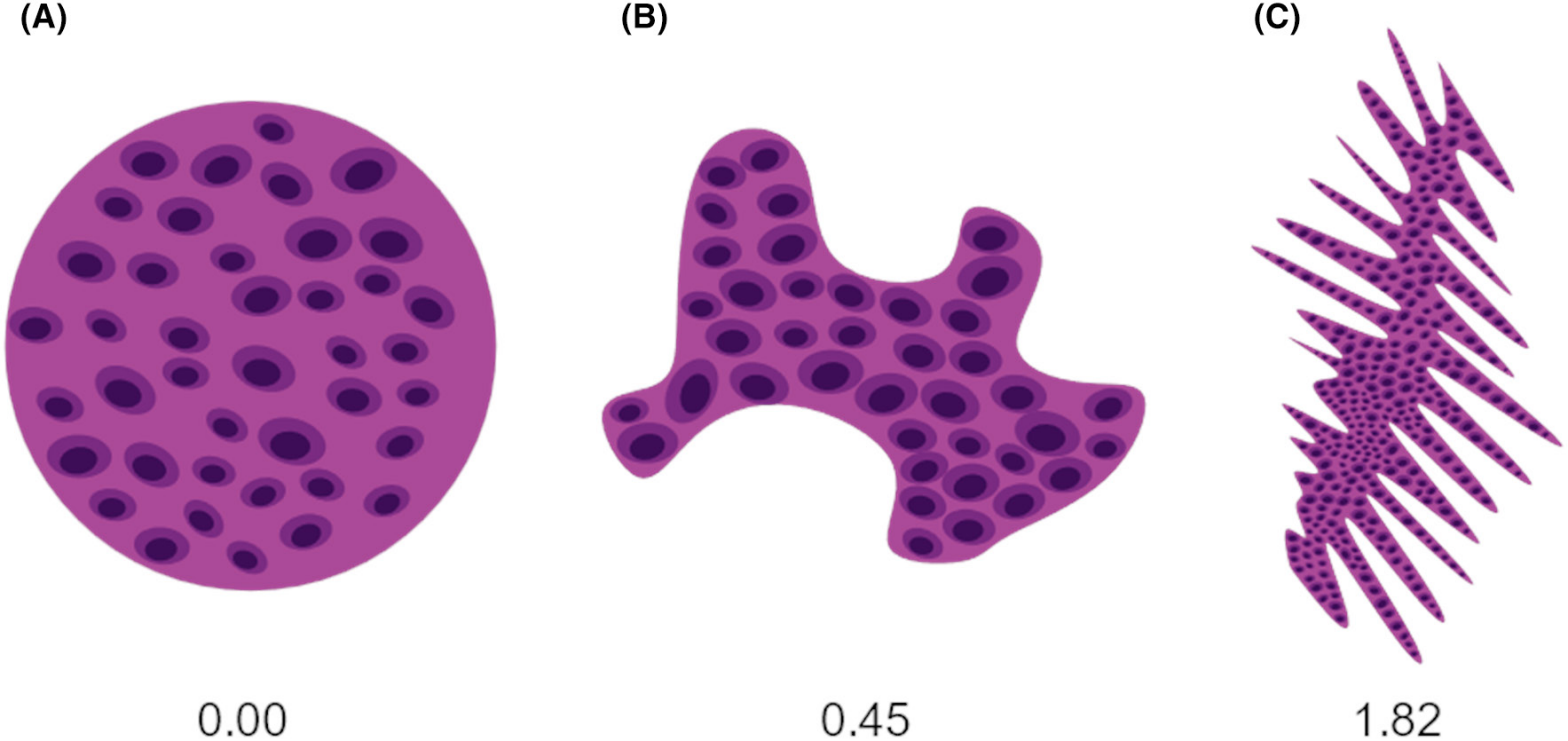
(A–C) Examples of different shapes with different modified Polsby–Popper (MPP) scores.

Téglási et al. characterized the growth patterns of brain metastases by manually delineating the infiltrative front of tumors in visual fields of a given width and comparing the tumor front length/visual window width‐ratio between cases.^32^ As an extension of this measurement method based on a pathomics approach, we included the total perimeter and total area of all annotations in each WSI which had been marked as cancerous compartments in the previously described semiautomated tissue detection. We hypothesized that a larger perimeter/area ratio of the tumor annotations of a WSI signifies a more undulating tumor front and the presence of a larger number of disjoint tumor islands. In this context, it was a logical step to search for a quantitative measure to describe this postulated phenomenon in a comparable fashion across slides. Subsequently, we started from the fact that for an area of a given size, the shape with the smallest possible perimeter is a circle, which is thus the most compact two‐dimensional shape. Polsby and Popper described the compactness of shapes in the light of the same mathematical consideration using the following equation:

(3) $$\mathrm{PP}=\frac{4\mathrm{\pi A}}{P^{2}},$$

where *A* is the area and *P* is the perimeter of the two‐dimensional region in question.^33^ The Polsby–Popper (PP) score, a dimensionless quantity, thus equals 1 for a perfectly circular region and tends to 0 for increasingly irregular regions. As our aim was to characterize the “waviness” of the tumor front and “disjointedness” of the tumor tissue compartment, we used the reciprocal of the original formula, causing any data calculated with the modified score to lie in the range [1,∞[, where 1 is the score for a perfectly circular region with a trend to infinity for increasingly irregular (more disjointed, “wavier”) regions. Furthermore, as our perimeter/area‐relationship dataset displayed a log‐normal distribution, logarithmic conversion was required to obtain normally distributed data and aid further statistical analysis.^34^ Based on these considerations, we created the following modified Polsby–Popper (MPP) score:

(4) $$\mathrm{MPP}=\frac{1}{2}\ln\frac{P^{2}}{4\mathrm{\pi A}},$$

where *A* is the area, and *P* is the perimeter of the two‐dimensional region in question. Consequently, we calculated the MPP score for the tumor annotations of each of the 100 WSIs. As quantitative measures retain a higher dimension of information than categorical values (notably the classification of the worst pattern of invasion according to Brandwein‐Gensler), we expected from introducing the MPP score (1) a more refined characterization of TSCC growth and invasion (2) eliminating interobserver variability as compared to qualitative assessment of tumor spread, and (3) higher information content for potential machine learning algorithms. Examples of MPP scores for different shapes are shown in Figure 3A–C.

### 2.4 Statistical analysis of the relationship between the MPP score and conventional histopathologic parameters and their effect on survival

Descriptive statistical analyses were performed and normality of data was assessed using the Shapiro–Wilk test, after which the appropriate correlation tests were performed to examine the relationship of the stroma/tumor ratio evaluated by the pathologist and by the computer‐assisted approach as well as the relationship between tumor thickness and MPP score. A correlation coefficient (*r*‐value) equal to or greater than 0.7 was interpreted as a strong positive correlation. As the results of the parametric and nonparametric tests were congruent, we report only those of the parametric tests. MPP scores of tumors with compact and discontinuous infiltration patterns, as well as MPP scores of tumors with and without extracapsular spread of lymph node metastases, were compared using the two‐tailed independent *t*‐test. One‐way analysis of variance (ANOVA) was used, where MPP scores of more than two subgroups were compared, for example, in the case of tumor size (pT1‐pT4a), tumor differentiation (Grade (G) 1–3) and the presence of occult metastatic disease (no metastases present/occult metastases present/manifest metastases present as defined below), after which either a Tukey post hoc test (homogeneity of variances assumed) or a Games‐Howell post hoc test (homogeneity of variances not assumed) was applied.

When further analyzing resection margins in cases with a documented R0 resection according to the patient file, cases where a free margin equal to or more than 5 mm measured on the main specimen was achieved were labeled as “R0 free margin” (R0fm). Cases where a free margin of less than 5 mm measured on the main specimen was achieved were labeled as “R0 close margin” (R0cm).

Patients were considered to have occult cervical lymph node metastases if they showed no clinical evidence of nodal disease at the time of the primary surgery and no neck dissection was initially performed, but a regional relapse occurred within 2 years of tumor resection and an observation for at least 2 years after primary surgery was documented, or if they showed no clinical evidence of nodal disease at the time of the primary surgery, but an upfront neck dissection confirmed a pN+ status. Patients with suspicious lymph nodes in the preoperative setting and histopathologically confirmed lymph node involvement upon neck dissection were labeled as having manifest cervical metastases. Patients who underwent an upfront neck dissection and for whom a pN0 status was histopathologically proven as well as those who did not undergo primary neck dissection, but for whom an at least 2‐year observation without a locoregional relapse was documented were labeled as not having cervical metastases.

It is of note that, in some instances, we analyzed the effect of a distinct pNX category, stating that no simultaneous neck dissection was performed at the time of the initial ablative surgery of the primary tumor site.

The following oncological outcome measures given in months from the initial treatment modality were evaluated:

- overall survival (OS): time span from the day of the initial surgery until death or until the day of the last live follow‐up date, regardless of disease status.
- TSCC‐specific survival (TCSS): time span from the day of the initial surgery until TSCC‐related death or last live follow‐up date or date of death from other causes.
- relapse‐free survival (RFS): time span from the day of the initial surgery until the first histologically documented local (according to the Warren and Gates criteria^35^) or regional recurrence of the TSCC or last follow‐up date with disease‐free status.

In specific instances, importantly, when comparing the R0fm and R0cm subgroups, metastasis‐free survival (MFS) was also evaluated as the time span from the day of the initial surgery until the first histologically documented distant metastasis which could be unequivocally attributed to the TSCC or last follow‐up date with disease‐free status.

Patients lost to follow‐up were censored at the time of the last documented follow‐up date. All survival analyses were performed using the Kaplan–Meier method and compared using the log‐rank test. Further comparisons of survival among more than two groups were conducted using the post hoc chi‐squared test. Survival probabilities were calculated using the survival package in the R Statistical Software (version 4.2.0; The R Foundation for Statistical Computing).^36^ To facilitate the interpretability of the MPP score, we employed a categorization approach based on the 75th percentile as an arbitrary threshold. As a result, we identified two distinct groups: a high category encompassing MPP scores higher than 4.2 and a low category comprising MPP scores equal to or less than 4.2. Multivariable Cox regression analyses were performed to evaluate the impact of tumor size, nodal disease, MPP score, and tumor grade on OS and TCSS. Statistical analyses were performed using the Statistical Package for Social Sciences (SPSS) version 22.0 (IBM, Armonk, NY), and *p* < 0.05 was considered statistically significant and marked with an asterisk (*). Effect sizes were determined using Cohen's *d* for *t*‐tests and eta‐squared (*η* ^2^) for ANOVA analyses.

### 2.5 Development of predictive models

To assess the possible use of the MPP score, as well as infiltration and growth patterns, in the prognostication of tongue carcinoma‐related death within 5 years after primary surgery, we developed machine learning (ML) models using clinical and pathological variables reflecting the intrinsic biological characteristics of TSCC. The ML algorithms evaluated included logistic regression, random forest, naïve Bayes, and XGBoost (eXtreme Gradient Boosting). Data preprocessing and model development were performed using Orange, a Python‐based open‐source data mining software.^37^ Missing values were omitted from the data analysis. Given the limited amount of data available in our dataset, the ML models were trained with 90% of the data and tested using the remaining 10% of the data. To enhance the performance of our prediction models, we utilized 50 randomized training‐test splits to create and assess 50 machine learning models for each algorithm. Hyperparameters were optimized manually between each 50‐fold set. All hyperparameters are listed in Supplementary Material 2. Manual feature elimination was performed to determine the best model. We started with all relevant clinicopathologic features, including the MPP score, as well as growth and infiltration patterns, and iteratively removed the least important features based on their impact on the model performance. We repeated this process until we found the best performing model under the given conditions. This manual feature elimination process allowed us to identify the most important features for our prediction task and helped reduce overfitting. Then, to evaluate the impact of the MPP score as well as infiltration and growth patterns on the performance of our ML models, we excluded these parameters from our dataset and repeated the 50‐fold learning process and hyperparameter optimization for each algorithm as a control group. This allowed us to determine the extent to which the removed parameters affected model performance using pairwise comparisons of the area under the receiver operating characteristic curve (ROC AUC) between models via the algorithm proposed by Sun and Xu^38^ on the basis of the method described by DeLong et al.^39^ Calculations were performed in RStudio 2022.02.2 Build 485 using the pROC package.^40^ We also developed machine learning models to predict cervical lymph node involvement (pN+) using a similar approach. However, in this case, a 20‐fold learning process was used to train and optimize the models. Additionally, to retain a sufficient number of cases, we included cN+ patients in our investigation as well. We hypothesized that by doing so, we can reduce imbalance in the target data distribution and predict the target variable with a higher accuracy than by selecting cN0 and cNX cases only. Our analyses were assessed using the International Journal of Medical Informatics (IJMEDI) checklist to ensure methodological quality (Supplementary Material 3).^41^ Image analyses, statistical computations, and the development of ML models were performed on a computer with an AMD Ryzen 75800H processor with Radeon Graphics running at 3.20 GHz using 16 GB of RAM, running Windows 11 Home.

## 3 RESULTS

### 3.1 Clinicopathologic characteristics

The clinicopathologic and treatment characteristics of our included patients are summarized in Table 1. Our study included 100 patients with a median age of 63.5 years (interquartile range 54.5–72.5 years). There were 34 females and 66 males. Neck dissection was performed in 76 cases, and 36 patients underwent reconstruction of the resection defect after ablative surgery; a radial forearm flap was used in 25 cases, followed by the anterolateral thigh flap in 7 cases, and other types of reconstructive techniques in 4 cases. According to the surgical pathology records, resection margins were classified as R0 in 95 of the 100 cases. A total of 14 patients had occult nodal disease, comprising 12 cN0/pN+ cases and 2 cases where a regional relapse occurred within a 2‐year observation time frame after primary surgery without upfront neck dissection. A more detailed enumeration of adjuvant therapy is provided in Table 2.

**TABLE 1:** Demographic and clinicopathologic characteristics of all patients (*n* = 100).

Variable	Value
Median age (years)	63.5 (IQR^a^ 54.5–72.5)
Sex (*n*)	
Male	66
Female	34
pT category	
pT1	40
pT2	39
pT3	17
pT4a	4
Tumor thickness (*n*)	
≤4 mm	24
>4 mm	76
pN category (*n*)	
pNX	24
pN0	45
pN1	8
pN2	20
pN3	3
Extranodal extension (*n*)	
Absent	61
Present	16
N/A^b^	23
Surgical margins (*n*)	
R0	95
R1	1
RX	2
N/A	1
Lymphovascular invasion (*n*)	
Absent	95
Present	4
N/A	1
Perineural invasion (*n*)	
Absent	66
Present	24
N/A	10
Tumor grading (*n*)	
Grade 1	9
Grade 2	51
Grade 3	39
N/A	1
Neck dissection (*n*)	
No	24
Yes	76
Defect reconstruction (*n*)	
No	64
Yes	36
Adjuvant therapy (*n*)	
No	40
Yes	60
Calculated median follow‐up (months)	93.6 (95% CI^c^: 77.7–108)
Maximum follow‐up (months)	185
Recurrence (*n*)	24
Regional	7
Local	16
N/A	1
Death (*n*)	54
Death by disease (*n*)	20

^a^ Interquartile range.

^b^ Not assessed, information not retrievable or does not apply.

^c^ Confidence interval.

**TABLE 2:** Adjuvant therapy details.

	Tumor size			
	pT1	pT2	pT3	pT4a
None	24	12	4	0
Brachytherapy	7	8	0	0
Percutaneous radiation	3	5	2	0
Chemotherapy	0	0	1	0
Percutaneous radiation + chemotherapy	4	10	9	4
Brachytherapy + chemotherapy	1	1	0	0
Brachytherapy + percutaneous radiation	0	2	1	0
Brachytherapy + percutaneous radiation + chemotherapy	1	1	0	0

### 3.2 The relationship of the MPP score with conventional clinicopathologic and histological features

**FIGURE 4:**
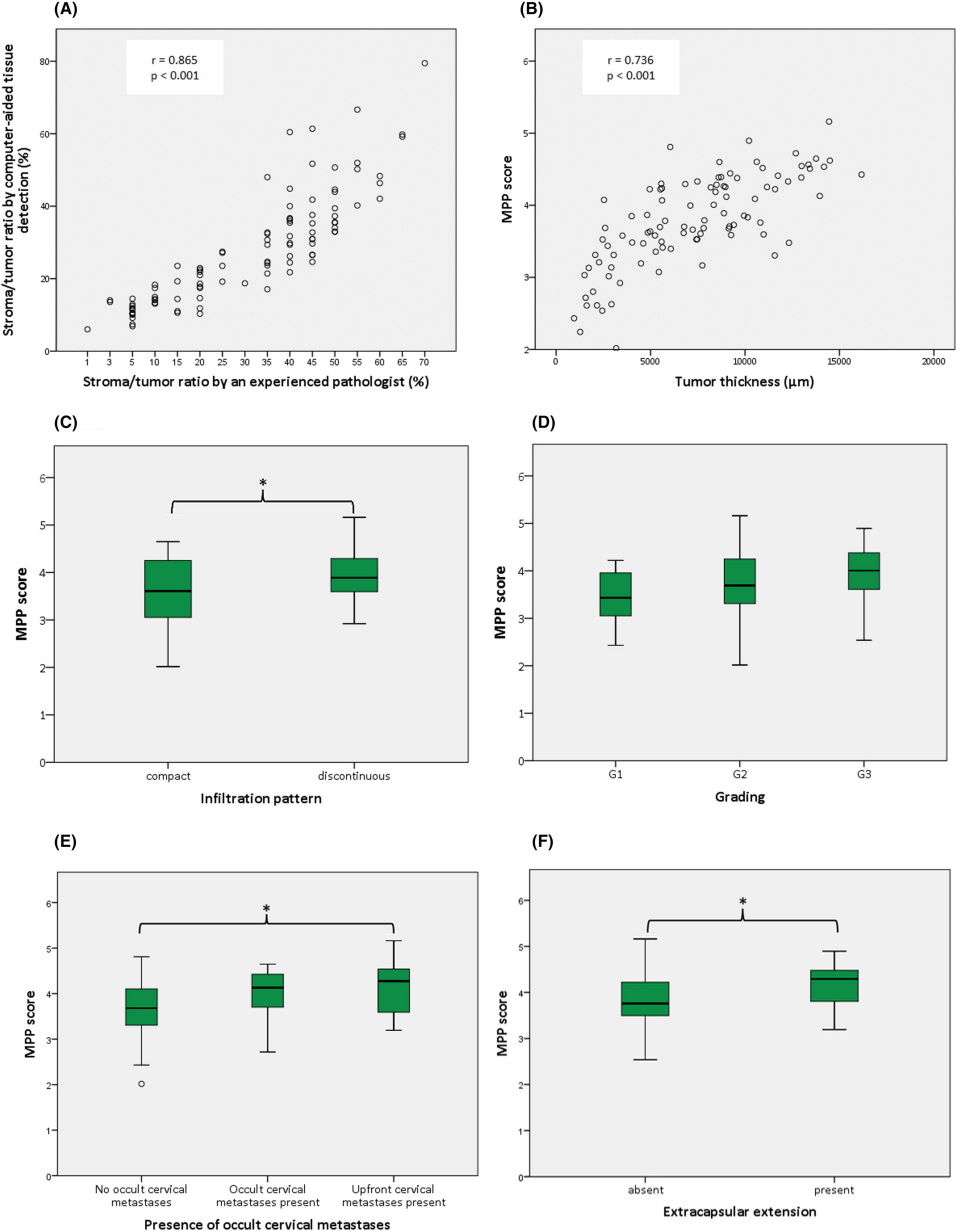
Quality control of the computer‐assisted tissue detection and the relationship of the modified Polsby–Popper (MPP) score with the evaluated conventional clinicopathologic parameters. (A) Assessment of stromal content by an experienced pathologist and by our computer‐based approach show a strong correlation. (B) MPP score and tumor thickness show a strong positive correlation. (C) Tumors with a discontinuous infiltration pattern exhibit significantly higher MPP scores than those with a compact infiltration pattern. (D) MPP score has no statistically relevant association with tumor differentiation grade. (E) MPP scores are significantly higher in cases with histologically proven cervical nodal involvement than in cases without nodal disease. (F) MPP scores are significantly higher where extracapsular spread of metastatic cells is present.

The stromal content in the central tumor assessed by the pathologist (Equation 1) and by the computer‐aided process (Equation 2) showed a strong correlation (*r* = 0.865, *p* < 0.001), suggesting satisfactory tissue discrimination of the semiautomated superpixel classification (Figure 4A).

As expected, tumor thickness showed significant differences across the pT categories (F(3,96) = 20.25, *p* < 0.01, *η* ^2^ = 0.39), with significantly higher tumor thickness values in pT4a (11,436 ± 2017 μm), pT3 (9267 ± 2549 μm), and pT2 cases (8776 ± 3446 μm) than in pT1 cases (4436 ± 2759 μm).

We then examined the relationship between tumor thickness and the MPP score, which showed a strong correlation between these variables (*r* = 0.736, *p* < 0.001; Figure 4B). MPP scores were significantly different in the respective pT categories (F(3,93) = 10.91, *p* < 0.001, *η* ^2^ = 0.26), and the Tukey post hoc test showed significantly higher MPP scores for pT4a, pT3, and pT2 tumors than for pT1 tumors (Table 3). Moreover, tumors with a discohesive growth pattern had significantly higher MPP scores than those with a cohesive growth pattern when all pT categories were considered (*t*(95) = 3.996, *p* < 0.001, *d* = 0.821). Similar results were observed when comparing the MPP scores of tumors with different infiltration patterns with a discontinuous infiltration pattern associated with higher MPP scores compared to tumors with a compact infiltration pattern (*t*(72.02) = 2.83, *p* = 0.006, *d* = 0.599; Figure 4C). Moreover, we found significant MPP score differences among the respective tumor differentiation grades (F(2,94) = 3.83, *p* = 0.025), but the Games‐Howell post‐hoc test failed to reveal a statistically significant difference between specific pairs (Figure 4D). Importantly, however, MPP scores were significantly different according to the presence of nodal metastases (F(2,90) = 5.5, *p* = 0.006, *η* ^2^ = 0.109), and the Tukey post hoc test revealed significantly higher MPP scores in cases where manifest nodal disease was present at the time of diagnosis compared to patients without nodal disease who remained nodal disease‐free for at least two years after surgical treatment of the primary tumor (*p* = 0.007, Figure 4E). Moreover, the MPP score was significantly higher in patients with nodal disease where extracapsular extension of the tumor infiltrated lymph nodes (ECE) was present than in those without ECE (*t*(74) = 2.32, *p* = 0.023, *d* = 0.669; Figure 4F).

**TABLE 3:** The relationship of the modified Polsby–Popper (MPP) score with conventional clinicopathologic and histological features.

Variable	MPP score^a^ (mean ± standard deviation)
pT category	
pT1	3.39 ± 0.65
pT2	4.04 ± 0.46
pT3	3.94 ± 0.54
pT4a	4.33 ± 0.42
Growth pattern	
Discohesive	3.99 ± 0.57
Cohesive	3.51 ± 0.62
Infiltration pattern	
Discontinuous	3.95 ± 0.49
Compact	3.59 ± 0.73
Extranodal metastatic spread	
Absent	3.8 ± 0.57
Present	4.17 ± 0.49
Presence of nodal disease	
No nodal disease	3.65 ± 0.60
Occult nodal disease	3.97 ± 0.64
Manifest nodal disease	4.13 ± 0.55
Tumor grading	
Grade 1	3.44 ± 0.62
Grade 2	3.69 ± 0.68
Grade 3	3.98 ± 0.63

^a^ Modified Polsby–Popper score.

### 3.3 Oncological outcomes

The median follow‐up time calculated using the reverse Kaplan–Meier estimator^42^ was 93.6 months (95% CI (confidence interval) 77.7–108 months). 24 patients developed recurrent disease, comprising 16 local and 7 regional recurrences; in one instance, localization was not retrievable. 54 patients died during follow‐up, and 20 deaths were unequivocally attributed to TSCC. The 5‐year OS was 62.6% (95% CI 53%–73%), the 5‐year TCSS was 83.5% (95% CI 76.2%–91.5%), and the 5‐year RFS was 74.9% (95% CI 66%–85.1%), respectively.

#### 3.3.1 Overall survival (OS)

**FIGURE 5:**
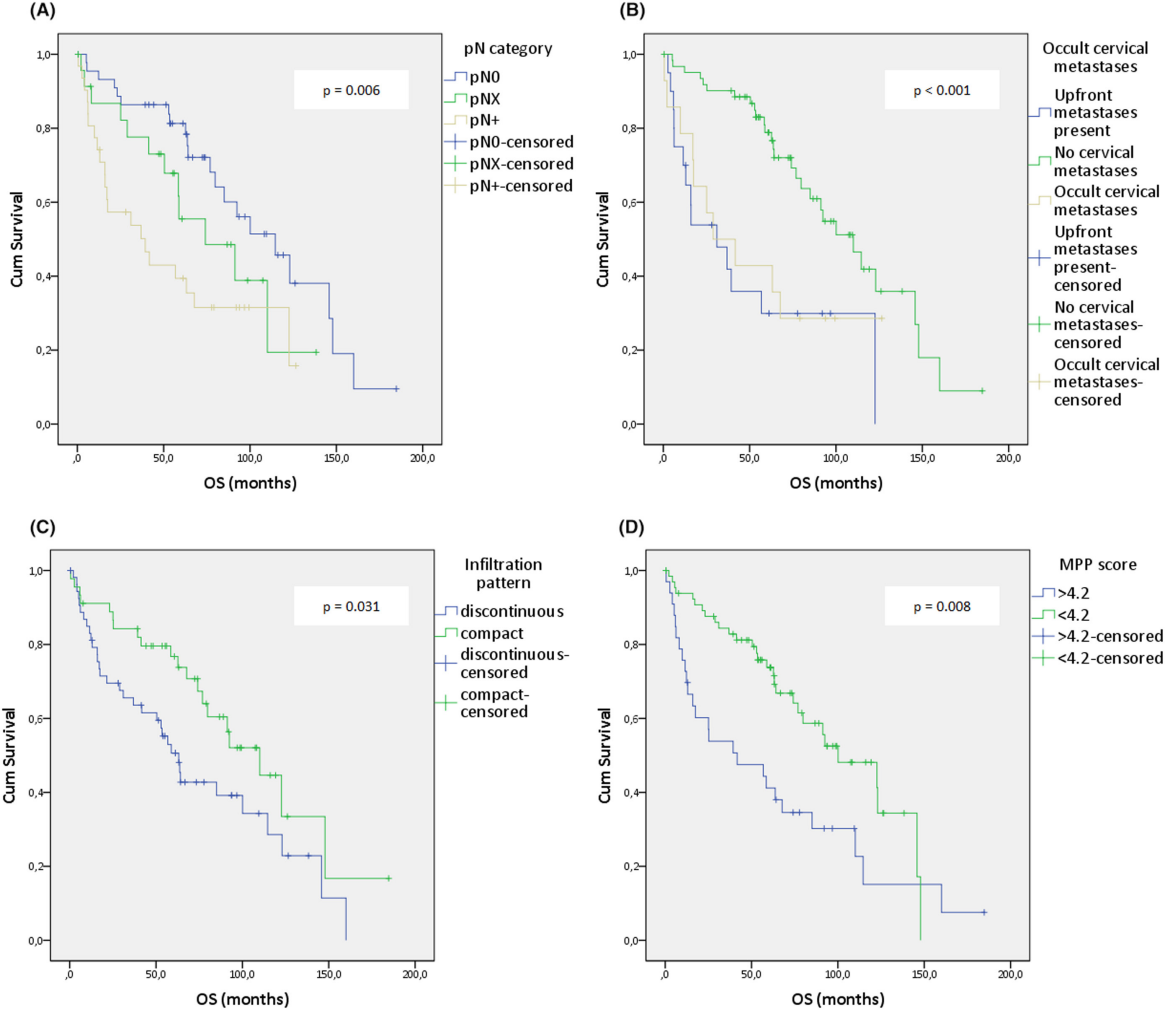
Kaplan–Meier analysis of selected clinical and pathological features on the overall survival of tongue squamous cell carcinoma (TSCC): (A) pathological N classification, (B) presence of occult cervical metastases, (C) infiltration pattern of the tumor, and (D) modified Polsby–Popper (MPP) score.

Higher pT categories, poorer tumor differentiation, tumor thickness >4 mm, and the presence of extracapsular extension were all found to be statistically linked to worse OS (logrank: *p* = 0.007; *p* < 0.001; *p* = 0.018; *p* = 0.003, respectively). Regarding nodal disease, we found a significant difference in survival among the groups pN0/pNX/pN+ (logrank: *χ* ^2^ = 10.29, df (degrees of freedom) = 2, *p* = 0.006), where a pathologically proven nodal involvement (pN+) had a significantly worse survival outcome than cases without nodal involvement (pN0) (post hoc: *χ* ^2^ = 10.16, *p* = 0.001). However, there was no significant difference in survival between cases with an unclear nodal status (pNX) and pN0 (post hoc: *χ* ^2^ = 2.41, *p* = 0.121) or between pNX and pN+ (post hoc: *χ* ^2^ = 1.65, *p* = 0.199; Figure 5A). When we examined the impact of occult nodal disease on OS by comparing cases with manifest, occult, or no nodal disease, we found a significant difference among the groups (logrank: *χ* ^2^ = 16.67, df = 2, *p* < 0.001). Importantly, both occult and manifest nodal disease showed similarly poor survival curves compared with no nodal disease (post hoc test for occult nodal disease: *χ* ^2^ = 7.12, *p* = 0.008; for manifest nodal disease: *χ* ^2^ = 15.37, *p* < 0.001). Moreover, there was no significant difference in survival between the occult and manifest nodal disease groups (post hoc: *χ* ^2^ = 0.24, *p* = 0.627; Figure 5B). Furthermore, tumors with a discontinuous infiltration pattern exhibited significantly worse survival than those with a compact infiltration pattern (logrank: *χ* ^2^ = 4.668, df = 1, *p* = 0.031; Figure 5C). Specifically, the 5‐year OS for patients with a discontinuous infiltration pattern was 50.7% (95% CI: 38.5%–66.7%), whereas for patients with a compact infiltration pattern, it was 76.8% (95% CI: 65.1%–90.6%; Table 4).

**TABLE 4:** Overall survival of all patients stratified according to the evaluated clinicopathologic features.

	Estimated median survival (months, 95% CI^a^)		2‐year overall survival (95% CI)		5‐year overall survival (95% CI)	
MPP score^b^ < 4.2	100.1	(65.2–135.1)	87.40%	(79.6%–96%)	73.50%	(63.1%–85.6%)
MPP score > 4.2	41.7	(0.0–84.9)	60.30%	(45.7%–79.7%)	41.30%	(27.3%–62.4%)
Tumor thickness < 4 mm	145.8	^c^	91.50%	(80.8%–100%)	84.90%	(70.2%–100%)
Tumor thickness > 4 mm	67.7	(47.1–88.2)	74.20%	(64.9%–84.9%)	55.50%	(45%–68.3%)
pT category						
1	100.1	(52–148.3)	89.80%	(80.8%–99.8%)	74.70%	(61.5%–90.7%)
2	67.7	(26.8–108.6)	75.70%	(63%–90.8%)	59%	(45%–77.4%)
3 and 4a	53	(27.4–78.5)	61.20%	(43.4%–86.4%)	43.70%	(26%‐73.4)
Nodal disease						
pN0	114.6	(81.5–147.7)	88.60%	(79.7%–98.5%)	81.30%	(70.4%–93.9%)
pNX	74.2	(28.6–119.7)	86.70%	(73.8%–100%)	55.50%	(37.1%–83.1%)
pN+	39.3	(8.9–69.8)	57.50%	(42.3%–78%)	39.50%	(25.2%–62%)
Tumor grading						
1	92.5	(60.3–124.6)	90%	(73.2%–100%)	90%	(73.2%–100%)
2	123.0	(64.5–181.5)	89.80%	(81.8%–98.7%)	72.30%	(60.5%–86.5%)
3	53.0	(25.2–80.7)	60.10%	(46.3%–78%)	42.30%	(28.8%–62.2%)
Extranodal metastatic spread						
Absent	16.1	(63.1–137.1)	85%	(76.4%–94.5%)	72.40%	(61.7%–85%)
Present	100.1	(13.3–18.9)	41.70%	(22.9%–75.8%)	34.70%	(17.3%–69.7%)
Occult cervical metastases						
Absent	110.0	(85.5–134.5)	91.80%	(85.2%–99%)	78.80%	(68.8%–90.3%)
Present	28.9	(0–59.4)	64.30%	(43.5%–95%)	42.90%	(23.4%–78.5%)
Infiltration pattern						
Compact	110.0	(77.7–142.3)	88.80%	(80.1%–98.6%)	76.80%	(65.1%–90.6%)
Discontinuous	63.1	(51.1–75.2)	69.60%	(58.2%–83.2%)	50.70%	(38.5%–66.7%)

^a^ Confidence interval.

^b^ Modified Polsby–Popper score.

^c^ Denotes a case in which the estimated value could not be computed as the Kaplan–Meier curve did not cross the threshold of 0.5 on the y‐axis.

We then assessed the effect of MPP score on OS in a dichotomous manner and found that patients with high MPP scores (i.e., >4.2) had a significantly worse survival compared to those with an MPP score <4.2 (logrank: *χ* ^2^ = 7.122, df = 1, *p* = 0.008; Figure 5D). The 5‐year OS rates for patients with an MPP score greater than 4.2 were found to be 41.3% (95% CI: 27.3%–62.4%), while patients with an MPP score below 4.2 exhibited a higher 5‐year OS rate of 73.5% (95% CI: 63.1%–85.6%). Notably, when considering pT1‐pT2 tumors exclusively, the discriminatory capacity of the MPP score in terms of survival probabilities remained consistent. Patients with an MPP score >4.2 had a 5‐year OS of 45.8% (95% CI: 29.7%–70.8%), whereas those with an MPP score <4.2 demonstrated a 5‐year OS of 77.9% (95% CI: 67.1%–90.4%). Table 4 shows the estimated median survival and survival probabilities according to different clinicopathologic features.

#### 3.3.2 Tongue carcinoma‐specific survival (TCSS)

**FIGURE 6:**
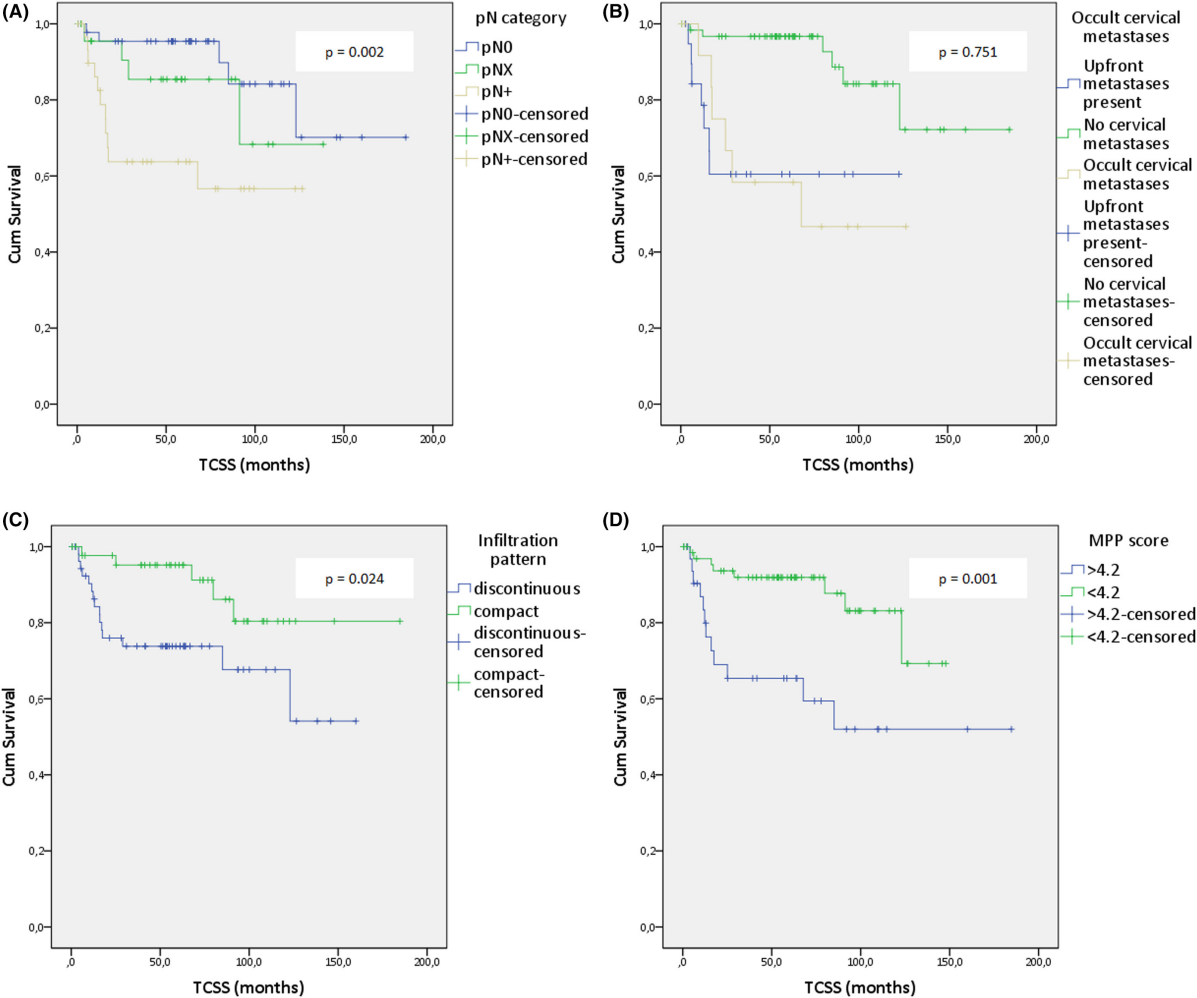
(A–D): Kaplan–Meier analysis of selected clinical and pathological features on the tongue carcinoma‐specific survival (TCSS) of tongue squamous cell carcinoma (TSCC): (A) pathological N classification, (B) presence of occult cervical metastases, (C) infiltration pattern of the tumor, (D) modified Polsby–Popper (MPP) score.

A higher pT category, poorer tumor differentiation, tumor thickness >4 mm, and the presence of ECE were statistically associated with worse TCSS (logrank: *p* < 0.001; *p* < 0.001; *p* = 0.008; *p* = 0.001, respectively). Similar to OS, the effect of nodal disease on TCSS was statistically significant (logrank: *χ* ^2^ = 9.97, df = 2, *p* = 0.007), and post hoc comparisons showed that the pN+ group had significantly worse survival than the pN0 group (post hoc: *χ* ^2^ = 9.41, *p* = 0.002), but there was no significant difference between pNX and pN0 (post hoc: *χ* ^2^ = 1.07, *p* = 0.302) or between pNX and pN+ (post hoc: *χ* ^2^ = 2.443, *p* = 0.118; Figure 6A). We found no statistically significant association between occult nodal disease and TSCC (logrank: *χ* ^2^ = 0.101, df = 1, *p* = 0.751; Figure 6B). Patients with a discontinuous infiltration pattern had significantly worse TCSS than those with a compact infiltration pattern (logrank: *χ* ^2^ = 5.125, df = 1, *p* = 0.024; Figure 6C). Furthermore, importantly, higher MPP scores were not only associated with worse overall survival outcomes but also significantly worse tongue carcinoma‐specific survival (logrank: *χ* ^2^ = 10.164, df = 1, *p* = 0.001; Figure 6D). These findings further support our hypothesis that utilizing the MPP score as a continuous parameter to describe tumor growth patterns can effectively describe the spectrum of biological aggressiveness. Table 5 lists the TCSS probabilities according to different clinicopathologic features.

**TABLE 5:** Two‐year and 5‐year tongue carcinoma‐specific survival of all patients stratified according to the evaluated clinicopathologic features.

	2‐year TCSS^a^ (95% CI^b^)		5‐year TCSS (95% CI)	
MPP score^c^ < 4.2	93.60%	(87.8%–99.9%)	91.90%	(85.4%–99%)
MPP score > 4.2	69.10%	(54.2%–88.2%)	65.50%	(50.2%–85.5%)
Tumor thickness < 4 mm	100%	(100%–100%)	100%	(100%–100%)
Tumor thickness > 4 mm	81.20%	(72.5%–91%)	78.20%	(69%–88.6%)
pT category				
1	97.30%	(92.2%–100%)	97.30%	(92.2%–100%)
2	85.40%	(74.4%–98.1%)	79.10%	(66.4%–94.2%)
3 and 4a	64.30%	(46.2%–89.5%)	64.30%	(46.2%–89.5%)
Nodal disease				
pN0	95.40%	(89.4%–100%)	95.40%	(89.4%–100%)
pNX	95.50%	(87.1%–100%)	85.40%	(71.4%–100%)
pN+	63.90%	(48.2%–84.6%)	63.90%	(48.2%–84.6%)
Tumor grading				
1	89.90%	(82.5%–97.9%)	89.90%	(82.5%–97.9%)
2	97.90%	(93.8%–100%)	93.30%	(86.3%–100%)
3	65.30%	(51.2%–83.3%)	65.30%	(51.2%–83.3%)
Extranodal metastatic spread				
Absent	89.90%	(82.5%–97.9%)	89.90%	(82.5%–97.9%)
Present	51.60%	(29.9%–89.1%)	51.60%	(29.9%–89.1%)
Occult cervical metastases				
Absent	96.70%	(92.3%–100%)	96.70%	(92.3%–100%)
Present	75%	(54.1%–100%)	58.30%	(36.2%–94.1%)
Infiltration pattern				
Compact	97.70%	(93.3%–100%)	95.20%	(88.9%–100%)
Discontinuous	76%	(65.1%–88.9%)	73.90%	(62.6%‐87.2)

^a^ Tongue carcinoma‐specific survival.

^b^ Confidence interval.

^c^ Modified Polsby–Popper score.

#### 3.3.3 Relapse‐free survival (RFS)

Our findings suggest that nodal status (i.e., pN0, pNX, or pN+), tumor grade (Grade 1, 2, or 3), tumor thickness (<4 mm or >4 mm), infiltration pattern (compact or discontinuous), and MPP score (<4.2 or >4.2) did not significantly affect RFS (logrank: *p* = 0.028 without significant differences in the pairwise post hoc tests; *p* = 0.319; *p* = 0.895; *p* = 0.605; *p* = 0.638, respectively), as we did not observe significant differences in RFS between the respective groups as mentioned above.

#### 3.3.4 Cox regression analysis on OS and TCSS

**FIGURE 7:**
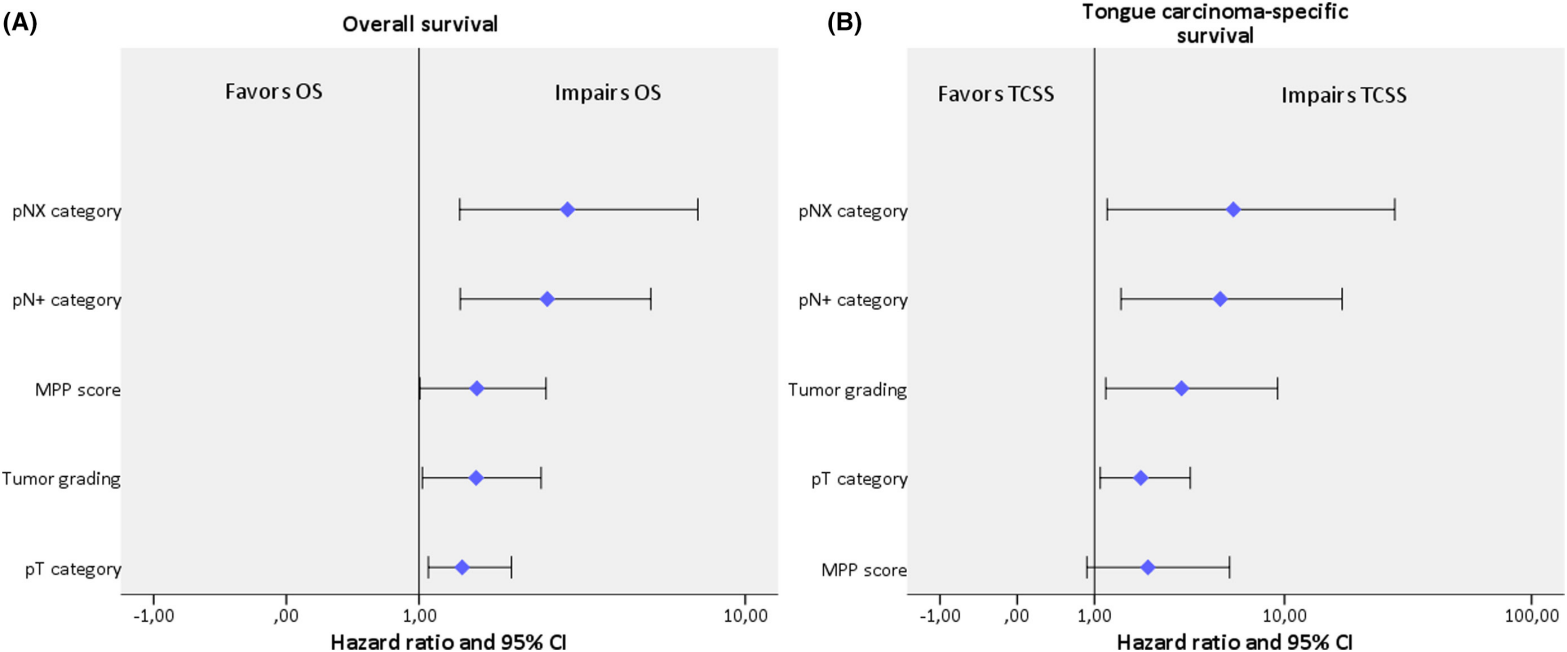
Forest plots of a Cox multivariate regression analysis on (A) overall survival (OS), (B) tongue carcinoma‐specific survival (TCSS) considering tumor size, nodal disease, tumor grade, and the modified Polsby–Popper (MPP) score.

Multivariate Cox regression analysis of the best evaluated parameters in univariate analysis revealed several significant associations with overall survival (OS). Notably, pT category (HR (hazard ratio) = 1.51, 95% CI: 1.011–2.243, *p* = 0.044), tumor grade (HR = 1.7, 95% CI: 1.035–2.784, *p* = 0.036), pNX nodal status (HR = 3.35, 95% CI: 1.475–7.592, *p* = 0.004), and the presence of manifest metastatic cervical lymph nodes at the time of diagnosis (HR = 2.91, 95% CI: 1.481–5.714, *p* = 0.002) were all found to be significant predictors of OS. Of particular importance is the fact that the MPP score (HR = 1.71, 95% CI: 1.009–2.884, *p* = 0.046) also exhibited a significant association with OS. This indicates that for each unit increase in the MPP score, the risk of death due to any cause increased by a factor of 1.71, holding all other covariates constant (Figure 7A).

In the Cox regression analysis in terms of TCSS, pT category (HR = 2.03, 95% CI: 1.107–3.725, *p* = 0.022), tumor grade (HR = 3.37, 95% CI: 1.213–9.348, *p* = 0.02), pNX nodal status (HR = 5.96, 95% CI: 1.241–28.582, *p* = 0.026), pN+ nodal status (HR = 5.18, 95% CI: 1.536–17.464, *p* = 0.008), and MPP score (HR = 2.23, 95% CI: 0.869–5.725, *p* = 0.096) were included again as predictors. While the MPP score did not reach statistical significance, the other three covariates were significantly associated with the outcome of interest (Figure 7B).

#### 3.3.5 Survival analyses in the R0 close margin (R0cm) subgroup

**FIGURE 8:**
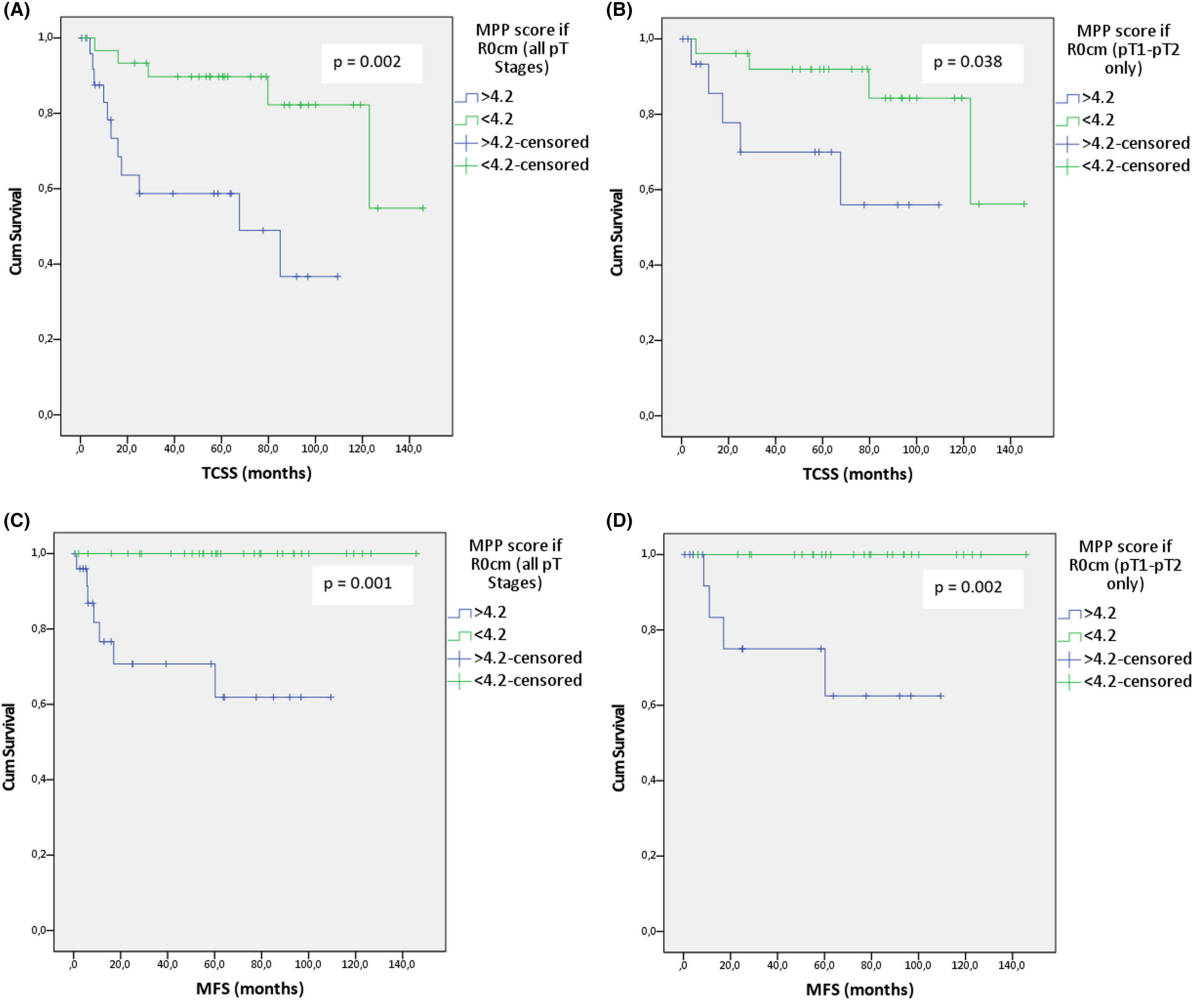
(A–D): Kaplan–Meier analyses of selected clinical and pathological features in the “R0 close margin” (R0cm) subgroup: the effect of the modified Polsby–Popper (MPP) score (A) on tongue carcinoma‐specific survival (TCSS) in all pT categories, (B) on TCSS in pT1‐pT2 categories only, (C) on metastasis‐free survival (MFS) in all pT categories, and (D) on MFS in pT1‐pT2 categories only.

As a next step, we focused our investigations on cases where the free resection margins of the main tumor specimen may not have reached 5 mm by excluding cases where a cancer‐free mucosal margin of ≥5 mm was unequivocally documented in the surgical pathology report; as mentioned above, we labeled the cases included as R0 “close margin” (R0cm). Survival analyses revealed that patients with an MPP score >4.2 had a significantly worse TCSS than those with an MPP score <4.2 (logrank: *χ* ^2^ = 9.749, df = 1, *p* = 0.002; Figure 8A; Table 6); moreover, this difference remained significant after excluding patients with pT3 and pT4 tumors (logrank: *χ* ^2^ = 4.296, df = 1, *p* = 0.038; Figure 8B). Although not statistically significant, an improved RFS could be observed in the group with an MPP score <4.2 (*p* = 0.244), and patients who underwent upfront or delayed additional resection of the primary tumor site because of close resection margins assessed intraoperatively or after evaluating the FFPE tissue specimen exhibited a more favorable RFS, although the results failed to reach statistical significance (*p* = 0.264). Notably, however, the MPP score had a good discriminatory capacity in the R0cm subgroup when assessing distant metastasis‐free survival (MFS), as all of our patients with MPP scores <4.2 remained free of distant metastases during the study observation period (logrank: *χ* ^2^ = 11.899, df = 1, *p* = 0.001; Figure 8C). This finding is particularly noteworthy since this statistical difference in MFS remains unchanged after excluding pT3 and pT4 tumors (logrank: *χ* ^2^ = 9.626, df = 1, *p* = 0.002; Figure 8D). In contrast, a tumor thickness of >4 mm versus <4 mm failed to demonstrate a statistically significant difference in terms of MFS in the Kaplan–Meier analysis, both when considering all cases (logrank: *χ* ^2^ = 2.782, df = 1, *p* = 0.095) and after excluding pT3 and pT4 TSCCs (logrank: *χ* ^2^ = 2.339, df = 1, *p* = 0.126).

**TABLE 6:** Five‐year relapse‐free survival, tongue carcinoma‐specific survival, and metastasis‐free survival according to modified Polsby–Popper (MPP) scores and additional resection status of the primary tumor site in the “R0 close margin” (R0cm) subgroup.

		5‐year relapse‐free survival (95% CI^a^)		5‐year tongue cancer‐specific survival (95% CI)		5‐year metastasis‐free survival (95% CI)	
MPP score^b^ < 4.2	For all pT categories	72.9%	(57.3%–92.7%)	89.7%	(79.4%–100%)	100.0%	(100%–100%)
MPP score > 4.2		60.5%	(41.9%–87.3%)	58.9%	(41.4%–83.9%)	70.7%	(53.3%–93.9%)
MPP score < 4.2	For pT1‐pT2	77.8%	(62.1%–97.4%)	92.0%	(81.9%–100%)	100.0%	(100%–100%)
MPP score > 4.2		65.6%	(43.2%–99.7%)	70.0%	(49.2%–99.7%)	75.0%	(54.1%–100%)
Additional resection	Performed	75.0%	(57.7%–97.4%)	86.4%	(73.2%–100%)	95.0%	(85.9%–100%)
	Not performed	61.9%	(45.5%–84.2%)	69.9%	(55.1%–88.6%)	83.2%	(70.7%–97.9%)

^a^ Confidence interval.

^b^ Modified Polsby–Popper score.

### 3.4 Prediction models

**FIGURE 9:**
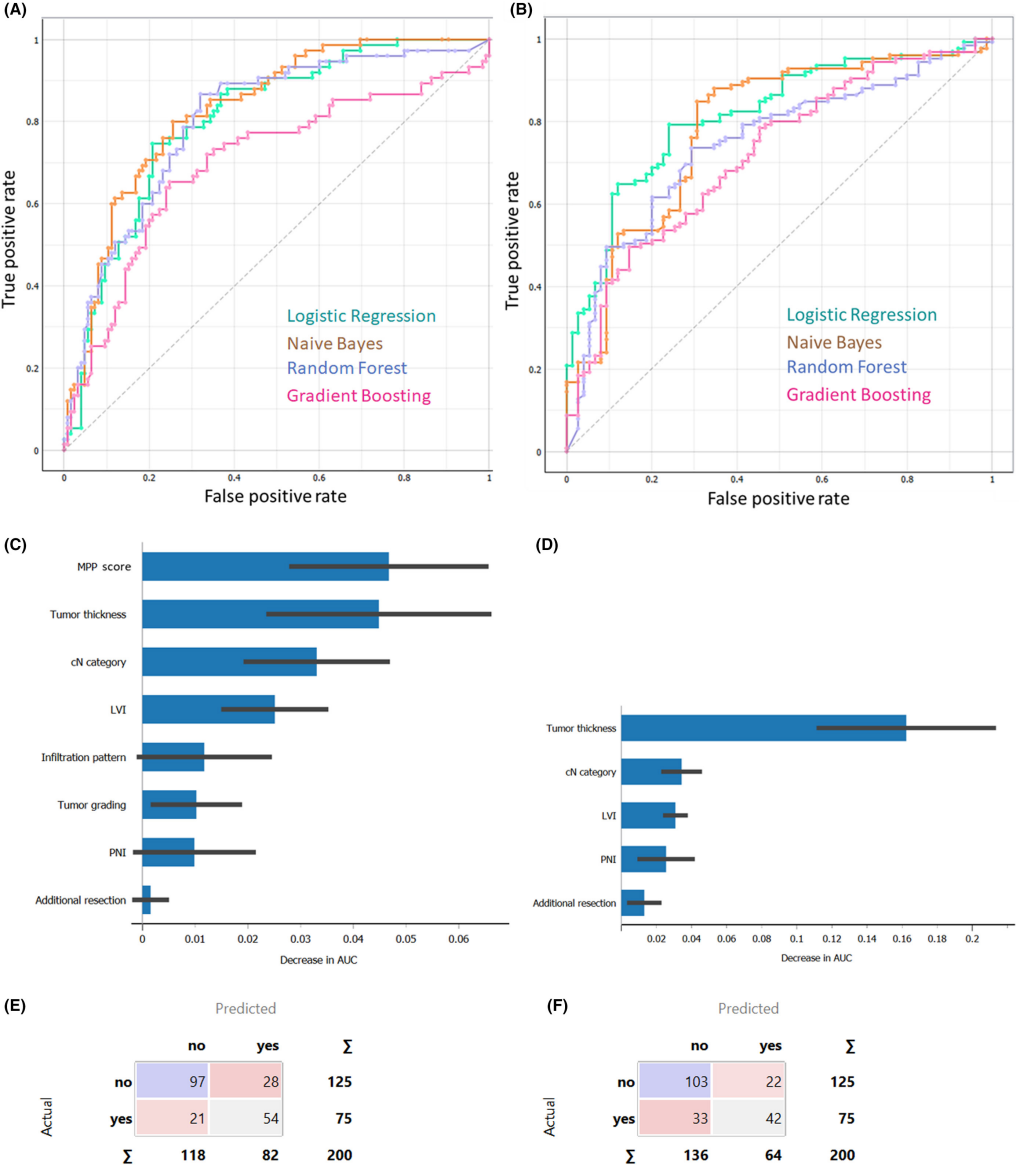
Predictive ability of machine learning models for lymph node positivity. (A) Area under the receiver operating characteristics (ROC) curves are shown for all four machine learning models when the modified Polsby–Popper (MPP) score and infiltration pattern included and (B) excluded. Relative feature importance of the best performing model when MPP score and infiltration pattern (C) included and (D) excluded. Confusion matrix of the best performing model when MPP score and infiltration pattern (E) included and (F) excluded.

**FIGURE 10:**
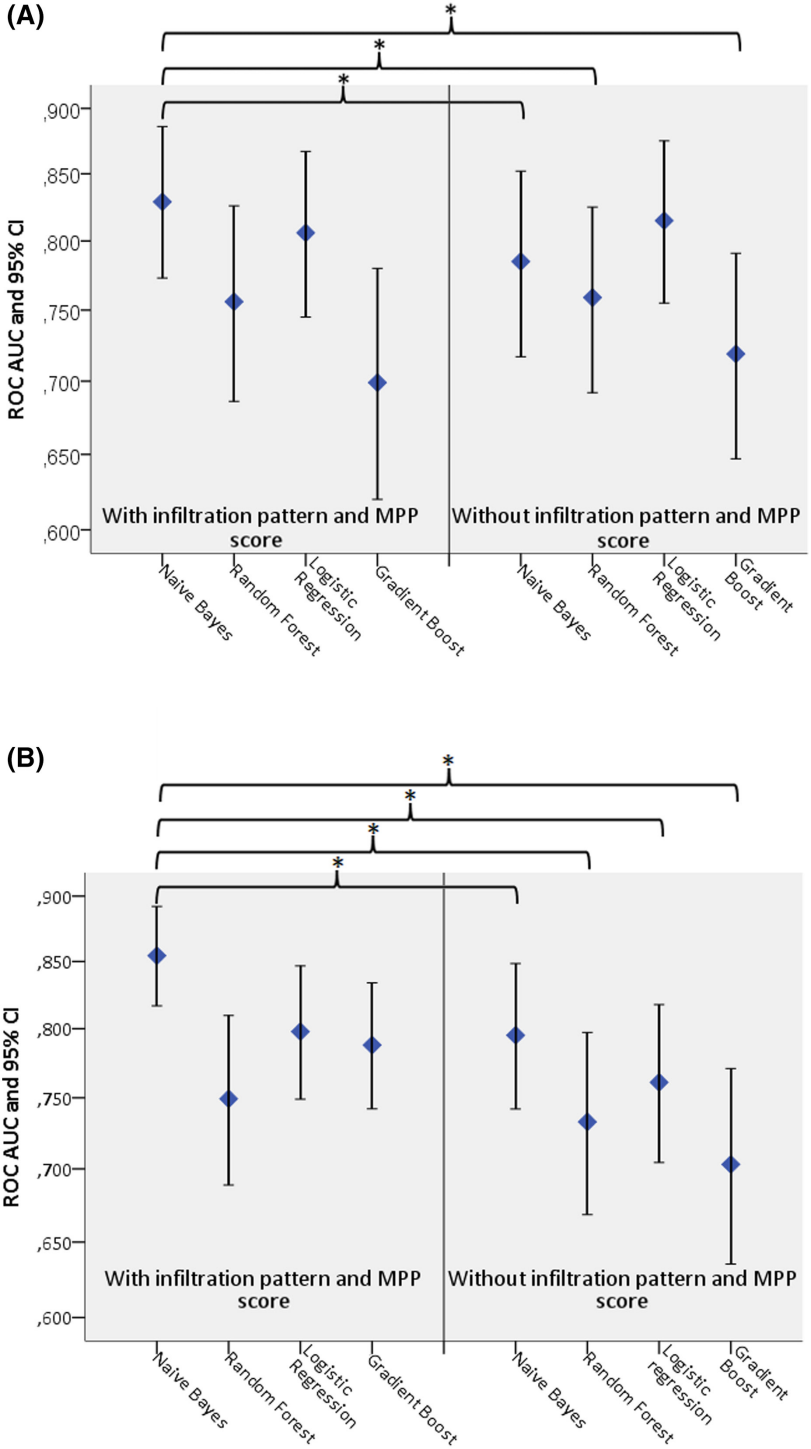
Pairwise comparisons of areas under the receiver operating characteristics (ROC) curves of different machine learning models (A) predicting nodal positivity when infiltration pattern and the modified Polsby–Popper (MPP) score included and excluded; (B) predicting 5‐years tongue carcinoma‐specific death when infiltration pattern, growth pattern as well as MPP score included and excluded.

Our study evaluated the performance of machine learning models in predicting occult cervical metastases in TSCC. The best performing model, that is, Naïve Bayes (Figure 9A) with MPP score and infiltration pattern included among the training features, found 42.3% of the occult metastases and identified the most important features as MPP score, tumor thickness, preoperative cN category, presence of lymphovascular invasion, and infiltration pattern (Figure 9C). Misclassification of patients without nodal disease was low, with only 22.4% of the patients being misclassified (Figure 9E). The model demonstrated a positive predictive value (PPV) and an area under the curve (AUC) of 0.761 and 0.829 (95% CI: 0.773–0.886), respectively. Interestingly, when the MPP score and infiltration pattern were excluded from the training process, a logistic regression model (Figure 9B) found 46.2% occult metastases, with only 17.6% of patients without nodal disease misclassified (Figure 9F). The most important features were tumor thickness, cN, LVI, PNI, and upfront or delayed additional resection of the primary tumor site (Figure 9D). This ML model had a PPV of 0.719, a sensitivity of 0.725, and an AUC of 0.815 (95% CI: 0.755–0.875), respectively. However, Naïve Bayes showed a significant improvement in performance when the MPP score and infiltration pattern were included compared to when these features were excluded (ROC AUC = 0.785, 95% CI: 0.717–0.852, DeLong test *p* = 0.0088, Figure 10A). In fact, of the 16 pairwise DeLong tests performed between ML models, 8 out of 16 model pairs showed a significantly increased ROC AUC when the MPP score and infiltration pattern were considered.

**FIGURE 11:**
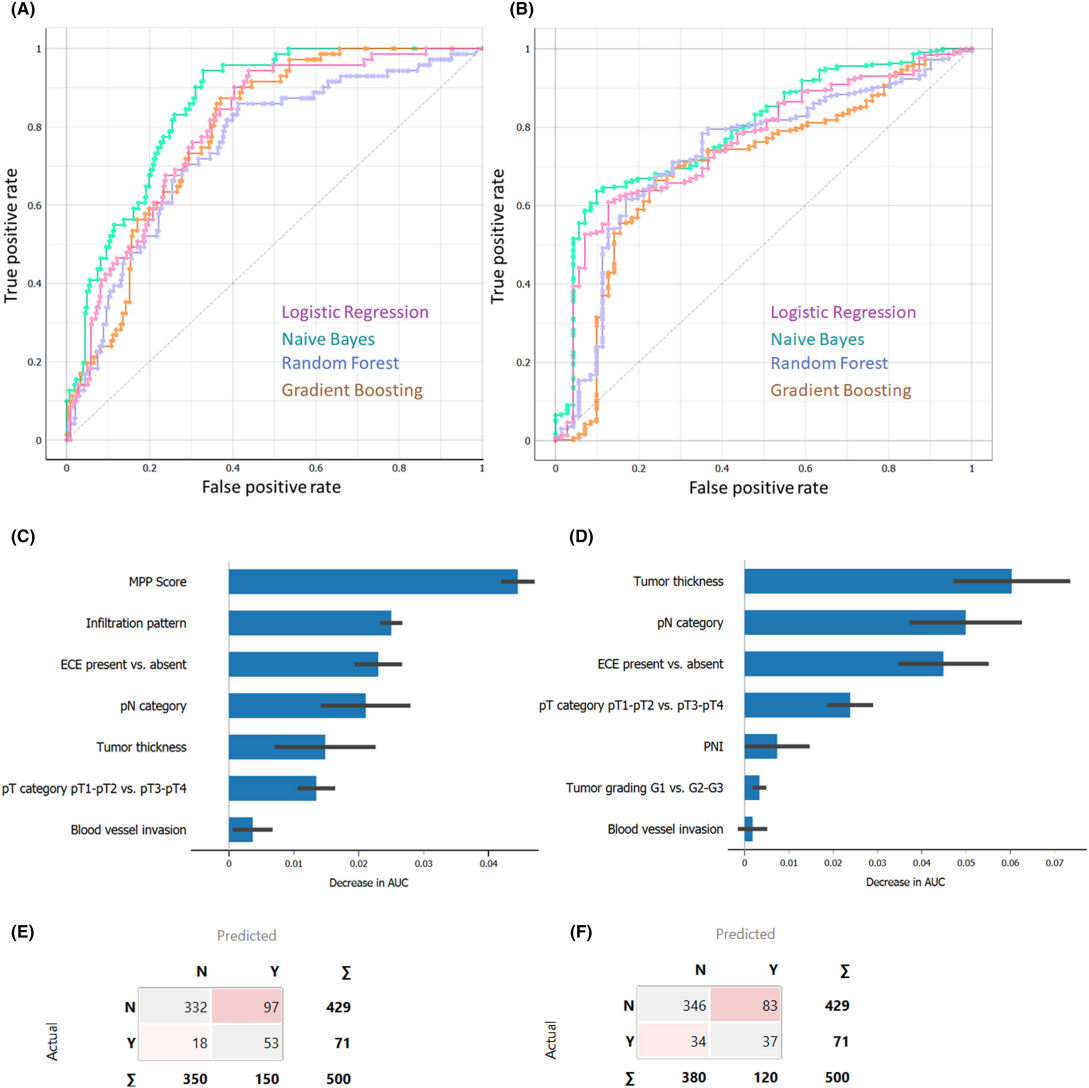
Predictive ability of machine learning models for tongue carcinoma‐specific death 5 years after surgical treatment. (A) Area under the receiver operating characteristic curves are shown for all four machine learning models when the modified Polsby–Popper (MPP) score as well as growth and infiltration pattern included and (B) excluded. Relative feature importance of the best performing model when MPP score and infiltration pattern (C) included and (D) excluded. Confusion matrix of the best performing model when MPP score and infiltration pattern (E) included and (F) excluded.

In this study, the prediction of 5‐year TSCC was investigated. When the MPP score as well as growth and infiltration patterns were included, the best model was found to be Naïve Bayes with a ROC AUC of 0.854 (95% CI: 0.817–0.892), a PPV of 0.864, and a sensitivity of 0.770. The most important features in this model were the MPP score, infiltration pattern, presence of ECE, pN category, and tumor thickness (Figure 11A,C,E). When the MPP score and growth and infiltration patterns were excluded, Naïve Bayes was still the best model with an ROC AUC of 0.765 (95% CI: 0.742–0.848), a PPV of 0.825, and a sensitivity of 0.766. The most important features in this model were tumor thickness, pN category, presence of ECE, pT category, and PNI (Figure 11B,D,F). Further analysis revealed that out of the 16 pairwise comparisons of the ROC AUC, the DeLong test showed a statistically significant increase in the ROC AUC in 9 of the possible 16 ML model pairs when the MPP score and infiltration pattern were considered (Figure 10B).

## 4 DISCUSSION

While Brandwein‐Gensler showed the significance of different tumor infiltration patterns almost two decades ago,^10^ these patterns play a minor role in today's clinical decision‐making. As H&E staining is the cornerstone of every histopathologic diagnostic process, it seems practical to extract as much relevant biological information as possible from H&E‐stained slides to facilitate personalized surgical therapy for TSCC. With the possibly growing number of patients to be treated, automatized diagnostic approaches are of emerging importance as well.

In this context, we developed an objective and quantifiable histological parameter, that is, the MPP score, describing the perimeter/area relationship of the tumor tissue compartment in H&E‐stained TSCC WSIs. We hypothesized that a greater MPP score would indicate more aggressive tumor biology, which may have an impact on survival and the presence of occult cervical metastases. Moreover, we examined the potential significance of the MPP score in cases where a close‐margin resection with tumor‐free borders of less than 5 mm (R0cm) was achieved. To ensure biological plausibility, we visually assessed growth and infiltration patterns in a dichotomous manner and found that the presence of a discohesive growth pattern and discontinuous infiltration pattern was associated with significantly higher MPP scores than cases with cohesive growth patterns and compact infiltration patterns. Additionally, our results showed that MPP scores were consistently higher across higher pT‐categories and were positively correlated with tumor thickness. Furthermore, MPP scores were significantly higher in cases with manifest nodal metastases and extracapsular extension (ECE). Taken together, these findings indicate that the MPP score may serve as a useful tissue biomarker to describe tumor aggressiveness in TSCC. In our study, we found that a higher pT category, poorer tumor differentiation, tumor thickness >4 mm, and presence of ECE were associated with worse OS and TCSS, as expected. Additionally, we observed that pN+ was significantly associated with worse OS and TCSS, which is consistent with previous reports. Furthermore, we found that pNX cases, albeit not statistically significant, may have worse OS and TCSS than pN0, highlighting the importance of detecting occult nodal disease. However, we did not find a statistically significant association between occult nodal disease and TCSS, possibly because of the low number of tongue cancer‐related deaths and our strict attribution criteria for cancer‐related deaths.

It is of note that we found MPP scores >4.2 to be associated with worse OS and TCSS in the univariate Kaplan–Meier analysis. Importantly, the MPP score is a useful metric for assessing survival in early TSCC. For example, in pT1‐pT2 tumors, patients with an MPP score >4.2 had a 5‐year OS of 45.8%, compared to 77.9% for patients with an MPP score <4.2. In the R0cm subgroup, we found that patients with an MPP score >4.2 had significantly worse TCSS than those with an MPP score <4.2 both when considering all pT categories and including pT1‐pT2 categories only. Notably, the MPP score was particularly useful in assessing distant metastasis‐free survival in cases where R0cm resection was achieved. All patients with MPP scores <4.2 remained free of distant metastases during the study observation period, while MPP scores >4.2 were associated with a 5‐year metastasis‐free survival of 70.7% for all pT categories and 75% for pT1‐pT2 TSCC. While our investigations did not show a statistically significant impact of the pN category, tumor grade, tumor thickness, infiltration pattern, or MPP score on RFS, our results support the assumption that an improved RFS of patients with close‐margin resections may exist if the MPP score is <4.2. Parallelly, although not statistically significant, patients may exhibit better survival after additional resection of the primary tumor site performed either during the same surgical procedure upon fresh frozen section assessment or in a delayed manner upon assessment of the FFPE tissue specimen.

Estimating life expectancy in a personalized manner has become increasingly important, as patients often want information about their survival chances, both qualitatively and quantitatively.^43^ Machine learning (ML) algorithms have outperformed traditional risk stratification models,^44^ prompting the development of ML models based on clinicopathologic parameters to predict prognosis in patients with oral cancer.^45^ In our study, the best‐performing ML model using conventional histopathologic parameters only showed that tumor thickness, pN category, and the presence of ECE were the most important features for predicting 5‐year TSCC‐related death. By comparing multiple ML model pairs, we found that including the MPP score and infiltration pattern data significantly improved the performance of most models. The best‐performing ML model with an additional MPP score and infiltration pattern data showed an outstanding predictive capacity for 5‐year TSCC‐related death, with an ROC AUC of 0.854, a PPV of 0.864, and a sensitivity of 0.770. In our study, we developed an ML model for predicting occult cervical metastases by incorporating the MPP score and infiltration pattern data. Our model demonstrated a PPV of 0.761 and an AUC of 0.829, with the most important features being the MPP score, tumor thickness, cN category, LVI, and infiltration pattern. Excluding the MPP score and infiltration pattern from the input parameters for the ML models resulted in tumor thickness, cN category, LVI, and PNI as the most important features.

The limited prognostic value of the traditional Broder grading system for head and neck squamous cell carcinoma (HNSCC) is well known. To address this issue, tumor budding (TB) has been examined as an alternative approach to assess tumor aggressiveness. Tumor budding is characterized by the presence of single tumor cells or small clusters of cells within the tumor center (intratumoral budding) or at the tumor invasion front (peritumoral budding).^46^ Stögbauer et al. developed a cellular dissociation grading system based on tumor budding and emphasized its relevance as an independent biomarker for adverse clinical outcomes both in human papillomavirus (HPV)‐positive and HPV‐negative HNSCC.^47^ Moreover, they demonstrated an increased probability of occult lymph node metastases, even when assessing only small tissue areas. In our study, the MPP score can be regarded as a metric that describes biological phenomena analogous to intratumoral and peritumoral budding, albeit using a pathomics approach. If a correlation between the MPP scores of excisional biopsies and the respective tumor specimens can be established, MPP scores could potentially be used to estimate tumor thickness based on small tumor tissue samples. This could result in better surgical planning and possibly a more accurate delineation of the target volume if primary radiochemotherapy is intended. Examining the MPP scores of biopsies is particularly important because of the potentially insufficient correlation between conventional histopathologic parameters of biopsies and surgical specimens.^48^ However, the relationship between the TB and the MPP score is beyond the scope of the present study, and the question of whether MPP scores of tumor biopsies could be used to guide treatment decisions, such as the need for elective neck dissection, remains to be explored in future studies.

Regarding RFS, several conclusions can be drawn. First, it would be crucial to gain a better understanding of the biology of TSCC relapse. Although the sample size in our investigation was relatively small, our results are consistent with those of previous studies that also failed to find a significant association between several conventional histopathologic parameters and RFS. Second, our findings suggest that patients with high MPP scores, particularly those with early stage TSCC, may benefit from tumor‐free margins >5 mm, even if additional surgical intervention is required. Importantly, this finding is indirectly in line with the work of Hakim et al., who also showed that postoperative radiotherapy did not improve any of the examined oncological outcomes in the R0fm and R0cm subgroups of pT1‐pT2 TSCC.^9^ However, as stated by Brandwein‐Gensler et al. too, it cannot be emphasized enough that inadequate resection margins have to be considered generally unacceptable; moreover, the same authors postulated that patients with low and intermediate histological risk patterns and inadequate surgical margins might benefit from re‐resection rather than adjuvant radiotherapy.^10^ Taken together, our investigations and other studies show that the adequacy of the resection margin cutoff values may have to be considered dynamically case‐dependent, as not only the infiltration depth but also a wide variety of parameters have to be taken into account.^49^ However, it is important to acknowledge that a considerable number of discrepancies can be observed across studies regarding this particular issue. For example, in Ref. [49], a general phenomenon applied to oral/pharyngeal cancers has been described, stating that with larger tumor extensions, the extent of microscopic infiltration of the surrounding healthy tissue also increases. Generally, this observation might hold true; however, the application of the MPP score would be instrumental in precisely defining a smaller patient group that could be at risk of recurrence and/or premature death, even in cases of seemingly adequate surgical clearance and/or early‐stage TSCC. Therefore, we advocate steering away from approaches that consider only a limited number of potentially important factors (e.g., as proposed in Ref. [50], suggesting redefining the adequacy of surgical margins based on primary tumor size). The contribution of such approaches to personalized oncological therapy might be debatable, as a limited number of attributes are not capable of predicting the target variable and they may overlook cases that can be deemed as “exceptions to the rule.”

From a broader perspective, the MPP score could play a key role in ensuring consistency in evaluating tumor spread across various institutions, effectively diminishing interobserver discrepancies. As already discussed above, there is considerable confusion regarding tumor growth patterns and the determination of appropriate surgical margins. The disparity in parameter relevance, such as the College of American Pathologists deeming tumor growth evaluation “redundant” and “optional,” while the ESMO considering it obligatory, adds to this complexity.^8^, ^11^ Furthermore, the categorical assessment of the WPOI in the Brandwein‐Gensler score system considering the tumor/host‐interface only may introduce biases and increase inter‐observer variability. Despite the ESMO guidelines mandating pathological assessment of tumor spread, the specific methodologies for assessment and defining adequate resection margins in a quantitative manner remain unspecified. By implementing the MPP score consistently across institutions, there is potential to analyze large, homogenous patient cohorts systematically. This standardized approach could lead to defining optimal resection margins based on given histopathologic parameters derived from surgical specimens.

Farrokhian et al. conducted a multicenter study to investigate the use of machine learning (ML) models to predict occult nodal metastases in 634 patients using clinical and pathological parameters as predictor variables.^21^ Their best‐performing model was the XGBoost model, with the most important parameters being those describing tumor behavior, namely LVI, grade, PNI, and DOI, resulting in an AUC of 0.838, a sensitivity of 0.927, and a PPV of 0.393. Two important implications emerge from the comparison between this study and our investigations. First, parameters regarding the biological behavior of TSCC and, most importantly, the MPP score can possibly serve as valuable inputs to improve the performance of ML models in predicting occult nodal metastases, even with a limited sample size. Second, meticulous preoperative staging is essential in assessing nodal status. In terms of predicting life expectancy, ML models targeting prognosis estimation in Ref. [45] achieved an ROC AUC of 0.7–0.75 and a sensitivity of 0.612–0.789. Our findings highlight the potential of ML models incorporating the MPP score and infiltration pattern data to improve the accuracy of predicting TSCC‐related death, providing valuable information for personalized patient counseling.

Our study has some limitations that should be acknowledged. First, the study sample of 100 patients was relatively small. However, we intentionally kept the cohort homogeneous by including only TSCC. This study serves as a proof‐of‐concept for the MPP score; however, larger datasets are needed to develop ML prediction worktools that can be used in clinical practice, as mentioned above. Second, our strict criteria for cancer‐related deaths may have artificially inflated TCSS probabilities, potentially leading to overestimation. In some instances, a larger sample size may have yielded lower values closer to the OS probabilities. Additionally, we used tumor thickness rather than infiltration depth in our investigation. This decision was based on our belief that measuring tumor thickness can be more easily automated in future investigations. Furthermore, many studies treat tumor thickness and infiltration depth as interchangeable, leading to inherent errors.^21^ However, we acknowledge that using only one diagnostic slide per case may have limited our ability to accurately determine the depth of invasion because the slide may not necessarily show the deepest invasion. Additionally, retrospective analysis of unoriented slides may have further complicated the measurement of the invasion depth. Another limitation of our study is that we intentionally excluded clinical data, such as age, smoking, and socioeconomic status. This was done because a retrospective review of patient records might not have provided sufficient data on these factors, and we wanted to focus strictly on parameters directly reflecting the biological behavior of TSCC.

There are naturally some application‐specific limitations to the use of the MPP formula. Since our scans are digital raster images, the measured perimeter and area will be dependent on resolution‐type parameters. However, because so‐called superpixels are used to determine and classify different regions, and the pixel size of the scans is much smaller than the superpixel border resolution, the pixel size will not affect the perimeter and area measurements. In turn, the superpixel size and the classification quality itself will affect these measurements, as well as the MPP score. This is partly because a smaller superpixel size would increase the measured area and perimeter but not necessarily at a rate that would keep the MPP score constant. Given the abovementioned factors that can influence the calculation of the MPP score, it is essential to standardize the processing and analysis of TSCC whole‐slide images. This is crucial to ensure the validity and comparability of MPP score results across different studies and institutions. Therefore, in future studies, we plan to focus on developing and implementing such a standardized pipeline to achieve reliable and consistent MPP score calculations.

The application of the MPP score is noteworthy as it presents opportunities for several future applications. In particular, the study of MPP scores of surgical biopsies would be of particular importance as a more in‐depth surgery planning, for example, detecting tumor‐free resection margins crossing non‐resectable anatomic structures or evaluating the need for microvascular reconstruction in the case of seemingly small TSCCs could be made possible. Moreover, determining MPP scores could pave the way for a more personalized patient education where different scenarios of surgical radicality and oncological outcomes could be discussed in the light of patient expectations in the pre‐therapy setting.

Taken together, this is the first study to establish the MPP score, a continuous variable reflecting tumor growth and infiltration features from a pathomics‐translational approach and its significance concerning the oncological outcome of TSCC with findings that should be validated in larger patient cohorts. As an ultimate goal, the MPP score can be further developed as a potentially automatable histological parameter aiding pathologist‐surgeon‐cooperation as an additional tool for therapy guidance in TSCC. However, additional steps of retrospective and prospective validation are required to fully elucidate the potential of the MPP score in TSCC and, in a broader sense, HNSCC.

## AUTHOR CONTRIBUTIONS

**Tamás Dániel Csűry:** Conceptualization (lead); data curation (lead); formal analysis (lead); investigation (lead); methodology (lead); visualization (lead); writing – original draft (lead). **Anna Zsófia Csűry:** Formal analysis (equal); methodology (equal); software (equal); visualization (equal); writing – original draft (equal). **Matthias Balk:** Supervision (equal); writing – review and editing (equal). **Andreas M. Kist:** Methodology (equal). **Robin Rupp:** Writing – review and editing (equal). **Sarina K. Mueller:** Writing – review and editing (equal). **Matti Sievert:** Writing – review and editing (equal). **Heinrich Iro:** Supervision (equal); writing – review and editing (equal). **Markus Eckstein:** Conceptualization (lead); data curation (lead); investigation (lead); methodology (lead); project administration (lead); supervision (lead); writing – review and editing (lead). **Antoniu‐Oreste Gostian:** Data curation (lead); project administration (lead); supervision (lead); writing – review and editing (lead).

## FUNDING INFORMATION

This study received no external funding.

## CONFLICT OF INTEREST STATEMENT

The authors declare no conflict of interest.

## ETHICAL APPROVAL STATEMENT

The study was conducted in accordance with the Declaration of Helsinki and approved by the Institutional Ethics Committee of the Friedrich‐Alexander University Erlangen‐Nuremberg (protocol code 23‐22‐Br). Informed consent was waived because of the retrospective nature of the study and the analysis used anonymous clinical data.

## Supporting information


**Supplementary Material S1:** Step‐by‐step description of the semiautomated image analysis pipeline using QuPath.

**Supplementary Material S2:** List of hyperparameters of the machine learning models assessed.

**Supplementary Material S3:** Checklist of the International Journal of Medical Informatics (IJMEDI) to ensure methodological quality of the machine learning models.

## Data Availability

Data are available on request from the corresponding author.
