# Supplementary material for: The modified Polsby–Popper score, a novel quantitative histomorphological biomarker and its potential to predict lymph node positivity and cancer‐specific survival in oral tongue squamous cell carcinoma
Source: Cancer Med. 2023 Dec 22;13(1):e6824. doi: 10.1002/cam4.6824 (PMC10807609; doi:10.1002/cam4.6824)
Supplement: Supplementary file 1 — Supplementary Material S1: Step‐by‐step description of the semiautomated image analysis pipeline using QuPath. [file CAM4-13-e6824-s002.pdf]

QuPath workflow

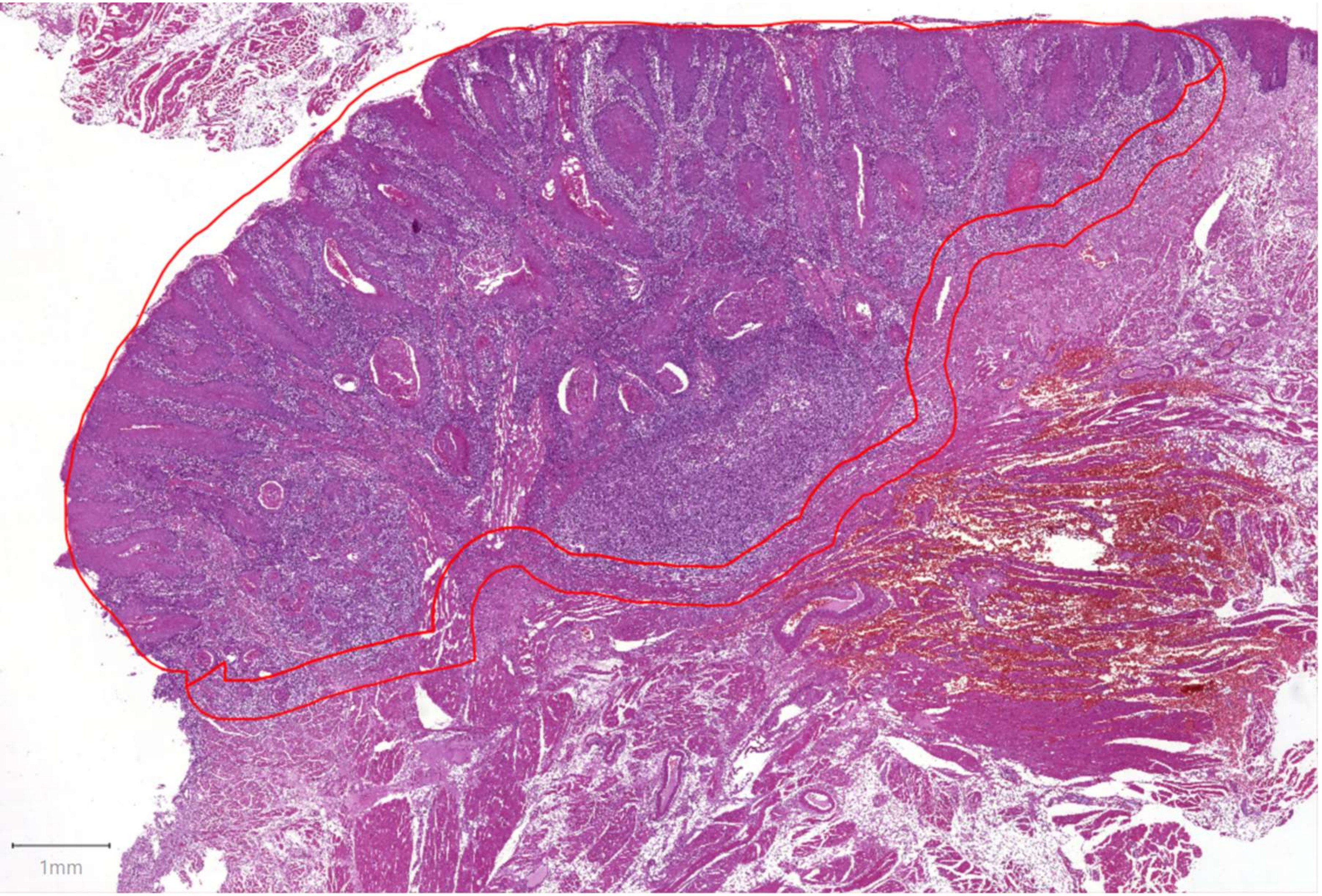

1. Annotation of the central tumor and peritumor area

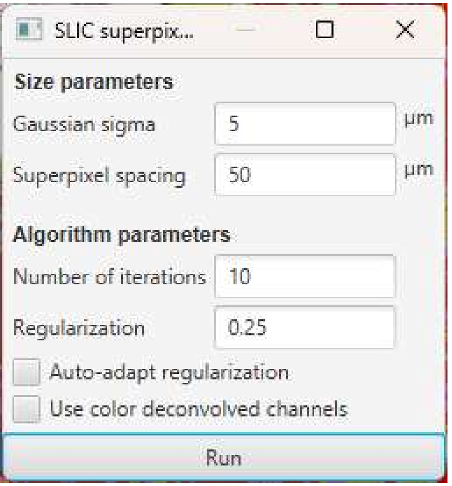

2. Superpixel segmentation with preset algorithm parameters

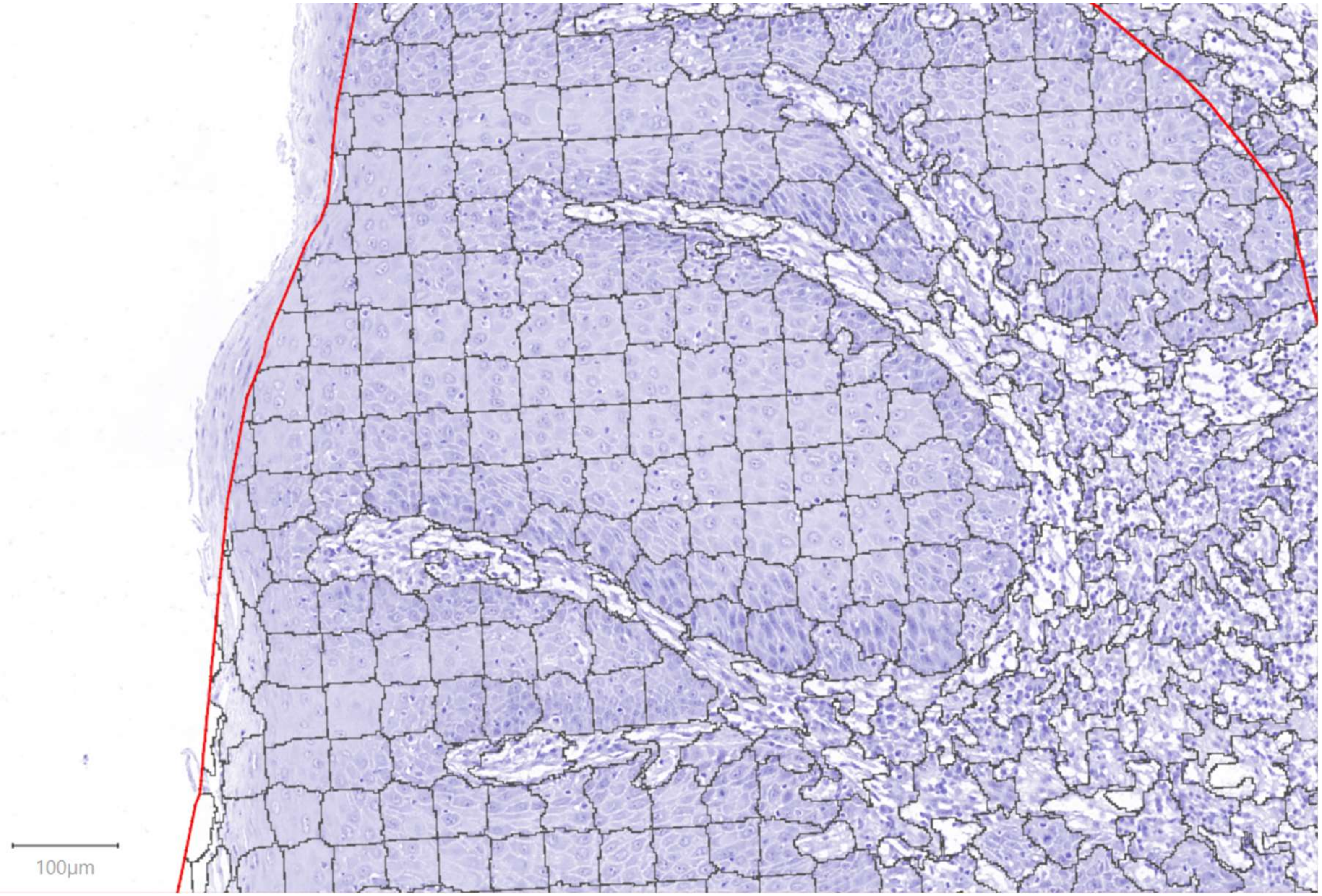

In this manipulated picture, we can see that the superpixel borders follow the border of the tumor nests in a satisfactory fashion.

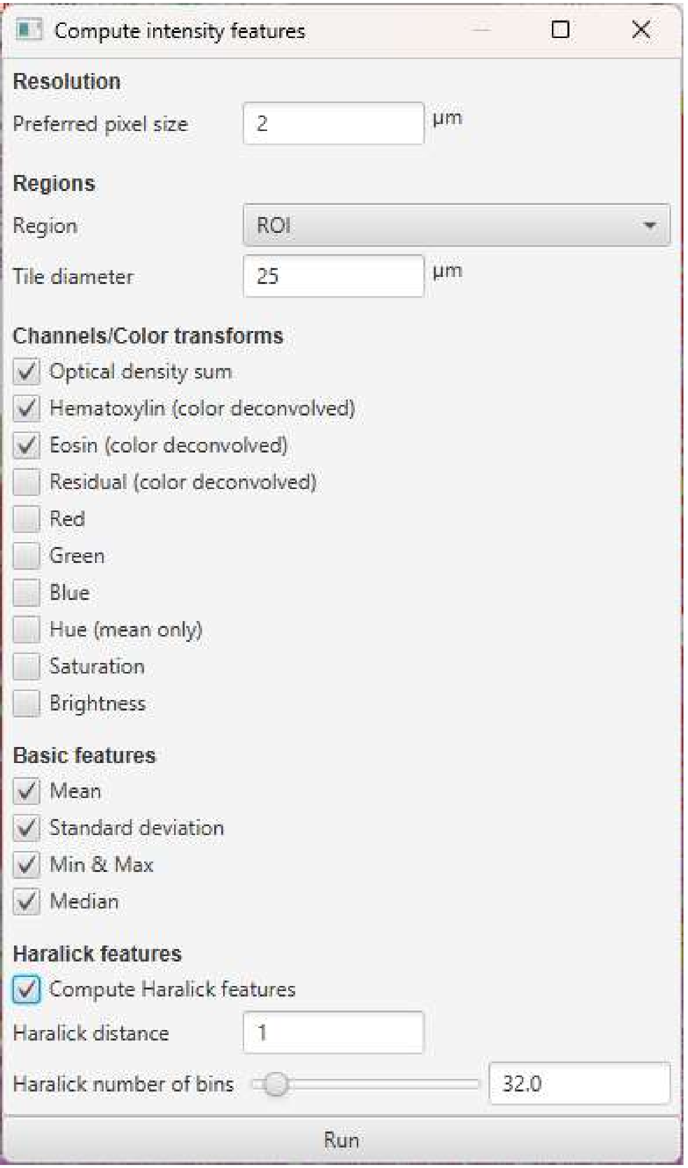

3. Intensity features of the superpixels were calculated.

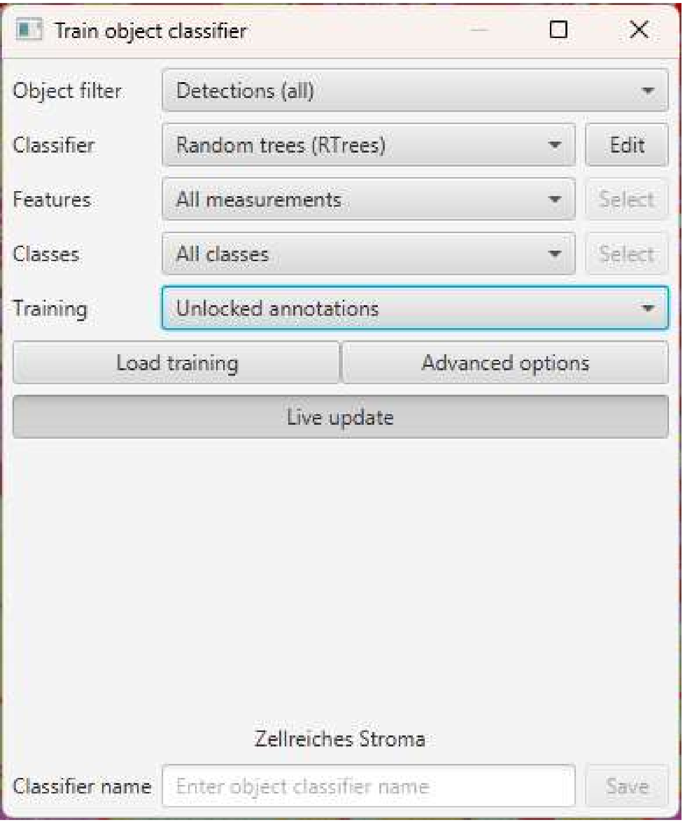

4. Classification of the superpixels was done in a semiautomated, visually controlled way by adding new annotations representing different tissue compartments until a satisfactory classification regarding the WSI was reached.

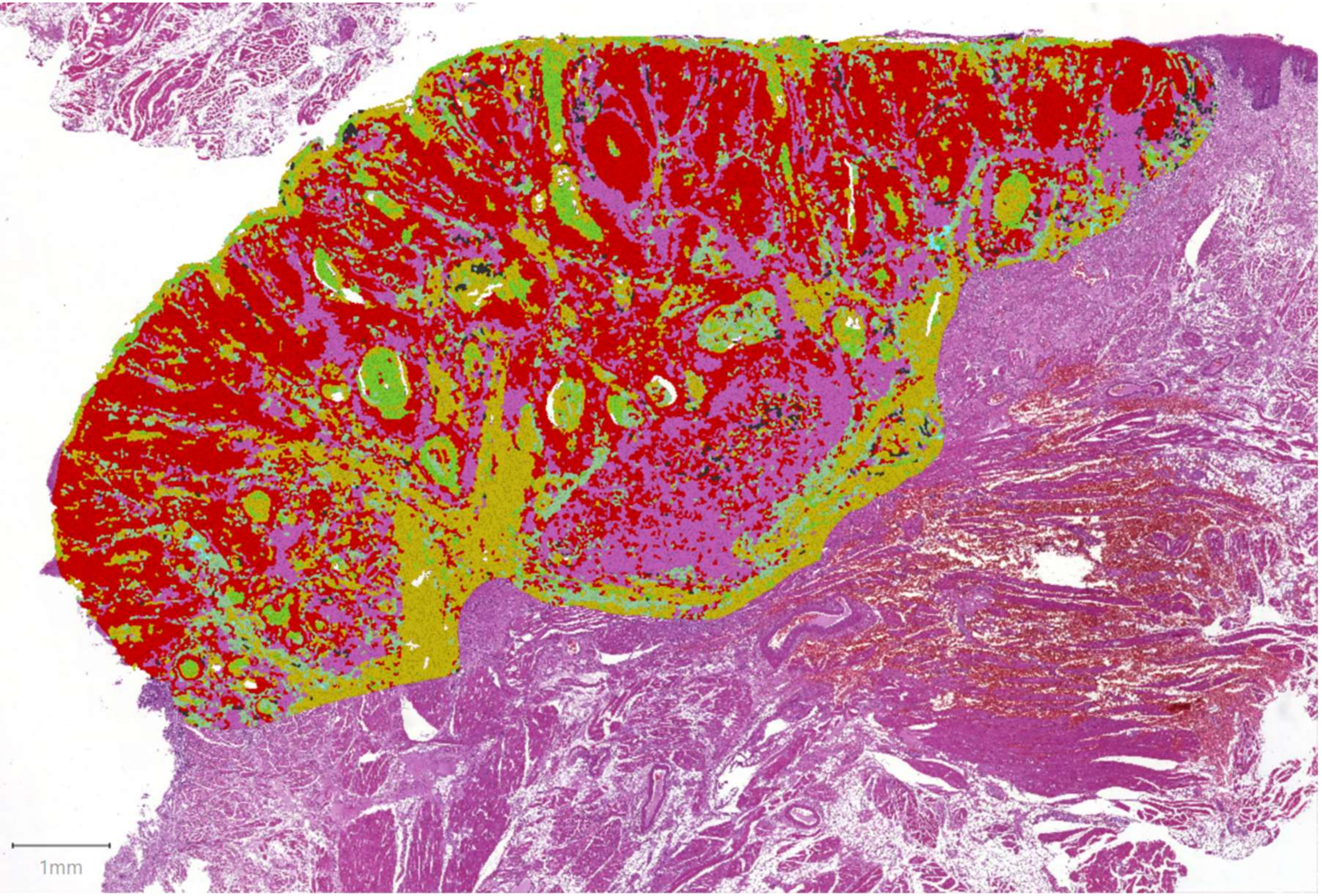

4. Classification of the superpixels was done in a semiautomated, visually controlled way by adding new annotations representing different tissue compartments until a satisfactory classification regarding the WSI was reached. We can clearly recognize the architecture of the tumor with tumor nests, keratin plugs and infiltration of the musculature.

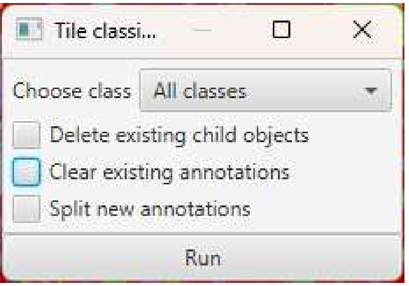

5. Superpixels classified as tumor tissue were then merged into a single tumor annotation to facilitate the extraction of perimeter and area measurements in terms of the whole tumor visible in a WSI.
