# Supplementary material for: The modified Polsby–Popper score, a novel quantitative histomorphological biomarker and its potential to predict lymph node positivity and cancer‐specific survival in oral tongue squamous cell carcinoma
Source: Cancer Med. 2023 Dec 22;13(1):e6824. doi: 10.1002/cam4.6824 (PMC10807609; doi:10.1002/cam4.6824)
Supplement: Supplementary file 2 — Supplementary Material S2: List of hyperparameters of the machine learning models assessed. [file CAM4-13-e6824-s003.pdf]

Prediction	Model	Tuning parameters and values
5-years tongue carcinoma-specific survival using MPP Score and infiltration + growth pattern	Logistic Regression	• Regularization type: L1 regularization, C = .09
	Random Forest	• Number of trees: 7<br>• Replicable training: On<br>• Maximum depth of individual trees: 3<br>• Minimum size of subsets for splitting: 5
	Naive Bayes	-
	XGBoost	• Number of trees: 60<br>• Learning rate: 0.3<br>• Replicable training: On<br>• Regularization: Lambda = 4<br>• Limit depth of individual trees: 3<br>• Fraction of training instances: 0.9<br>• Fraction of features for each tree: 0.9<br>• Fraction of features for each level: 0.95<br>• Fraction of features for each split: 1.00
5-years tongue carcinoma-specific survival without using MPP Score and infiltration or growth pattern	Logistic Regression	• Regularization type: L1 regularization, C = .09
	Random Forest	• Number of trees: 9<br>• Replicable training: On<br>• Balance class distribution: On<br>• Maximum depth of individual trees: 2<br>• Minimum size of subsets for splitting: 3
	Naive Bayes	-
	XGBoost	• Number of trees: 50<br>• Learning rate: 0.3<br>• Replicable training: On<br>• Regularization: Lambda = 6<br>• Limit depth of individual trees: 3<br>• Fraction of training instances: 0.95<br>• Fraction of features for each tree: 0.90<br>• Fraction of features for each level: 0.95<br>• Fraction of features for each split: 0.95
pN+ using MPP Score and infiltration pattern	Logistic Regression	• Regularization type: L1 regularization, C = .07
	Random Forest	• Number of trees: 10<br>• Minimum size of subsets for splitting: 5
	Naive Bayes	• -
	XGBoost	• Number of trees: 30<br>• Learning rate: 0.2<br>• Replicable training: On<br>• Regularization: Lambda = 0.7<br>• Limit depth of individual trees: 8<br>• Fraction of training instances: 1.00<br>• Fraction of features for each tree: 1.00<br>• Fraction of features for each level: 1.00<br>• Fraction of features for each split: 1.00
pN+ without using MPP Score or infiltration pattern	Logistic Regression	• Regularization type: L1 regularization, C = .07
	Random Forest	• Number of trees: 10<br>• Minimum size of subsets for splitting: 5
	Naive Bayes	• -
	XGBoost	• Number of trees: 30<br>• Learning rate: 0.2<br>• Replicable training: On<br>• Regularization: Lambda = 0.7<br>• Limit depth of individual trees: 8<br>• Fraction of training instances: 1.00<br>• Fraction of features for each tree: 1.00<br>• Fraction of features for each level: 1.00<br>• Fraction of features for each split: 1.00
